# Mixed-quantum-dot solar cells

**DOI:** 10.1038/s41467-017-01362-1

**Published:** 2017-11-06

**Authors:** Zhenyu Yang, James Z. Fan, Andrew H. Proppe, F. Pelayo García de Arquer, David Rossouw, Oleksandr Voznyy, Xinzheng Lan, Min Liu, Grant Walters, Rafael Quintero-Bermudez, Bin Sun, Sjoerd Hoogland, Gianluigi A. Botton, Shana O. Kelley, Edward H. Sargent

**Affiliations:** 1 Department of Electrical and Computer Engineering, University of Toronto, 10 King’s College Road, Toronto, ON Canada M5S 3G4; 2 Department of Chemistry, University of Toronto, 80 St. George Street, Toronto, ON Canada M5S 3G4; 3 Department of Materials Science and Engineering, McMaster University, Hamilton, ON Canada L8S 4M1

## Abstract

Colloidal quantum dots are emerging solution-processed materials for large-scale and low-cost photovoltaics. The recent advent of quantum dot inks has overcome the prior need for solid-state exchanges that previously added cost, complexity, and morphological disruption to the quantum dot solid. Unfortunately, these inks remain limited by the photocarrier diffusion length. Here we devise a strategy based on n- and p-type ligands that judiciously shifts the quantum dot band alignment. It leads to ink-based materials that retain the independent surface functionalization of quantum dots, and it creates distinguishable donor and acceptor domains for bulk heterojunctions. Interdot carrier transfer and exciton dissociation studies confirm efficient charge separation at the nanoscale interfaces between the two classes of quantum dots. We fabricate the first mixed-quantum-dot solar cells and achieve a power conversion of 10.4%, which surpasses the performance of previously reported bulk heterojunction quantum dot devices fully two-fold, indicating the potential of the mixed-quantum-dot approach.

## Abstract (web summary)

Solution processed colloidal quantum dots are emerging photovoltaic materials with tuneable infrared bandgaps. Here, Yang et al. create a class of quantum dot bulk heterojunction solar cell via ligand design, enabling longer photocarrier diffusion lengths for greater photocurrent and performance.

## Introduction

Solution-processed photovoltaic devices harvest abundant solar energy for conversion into electrical power while maintaining low manufacturing costs compared to conventional crystalline semiconductor devices^1^. Among emerging materials for third-generation photovoltaics^2^, colloidal quantum dots (QDs) are of great interest in view of their size-dependent bandgap that allows efficient absorption across the broad solar spectrum^3^. Advances in surface passivation and device architecture have led to consistent increases in photovoltaic performance, beginning from <1% in 2005 to a recently certified record of 11.3%^4, 5^.

Photovoltaic performance can be increased via charge separation using p–n junctions that interpenetrate at the nanoscale^6^. In organic solar cells, the bulk heterojunction (BHJ) enables exciton dissociation into free carriers to avoid bimolecular recombination and thereby increase performance^7^. The BHJ is achieved via material phase separation at the nanoscale during film preparation. The BHJ concept has been explored in QDs as well with the goal of extending the carrier diffusion length and achieving device thicknesses comparable to optical absorption length. However, QD BHJ devices have so far shown solar power conversion efficiencies that reached at most half those of the best planar devices^8, 9^. These prior BHJ devices have relied on solid-state (a.k.a. place) ligand exchanges, and thus required two different QD materials for the donor and acceptor phases^8^. Relative enhancements in current density have indicated progress on carrier extraction, but a low fill factor revealed the inferiority of the complementary QD material (Bi_2_S_3_, ZnO, or CdSe) compared to state-of-the-art PbS QDs. Employing PbS QDs for both donor and acceptor phases has so far been impossible, since in a place exchange, the whole material stack is treated homogeneously.

In this study, we took the view that—by contrast—a mixed QD ink would be an ideal material platform in which to advance PV performance. Each component could be separately optimized in solution before the mixture is formed. Surface ligands could be used to tailor electron affinity, allowing the shifting of QD band energy levels to prepare a desired hetero-offset^10^, as well as interdot coupling and carrier mobility for electrons and holes separately. We therefore prototyped the mixed-quantum-dot solid for solar cell applications. This type of mixed solid is based on an ink that combines, and retains the independent surface functionalization of, a donor and an acceptor class of QDs. Spectroscopic studies prove that the donor and acceptor dots are chemically distinct, and that inter-dot interactions are stabilized through hydrogen-bonding among surface ligands. Device simulations predict the optimal ratio of donor:acceptor in the mixture in light of asymmetric hole:electron mobilities, and experimental results agree well with the predicted optimum. Photoluminescence of the mixture shows strong quenching relative to the pure donor and acceptor components, indicating efficient charge separation at the nanoscale interface. Elementally resolved electron microscopy indicates a uniform mixture of donor and acceptor in the extended QD solid. We fabricate the first mixed-quantum-dot ink solar cells and achieve a solar power conversion of 10.4%, two times as high as the performance of previously reported BHJ QD devices^8^.

## Results

### Preparation of mixed-quantum-dot solid

**Fig. 1:**
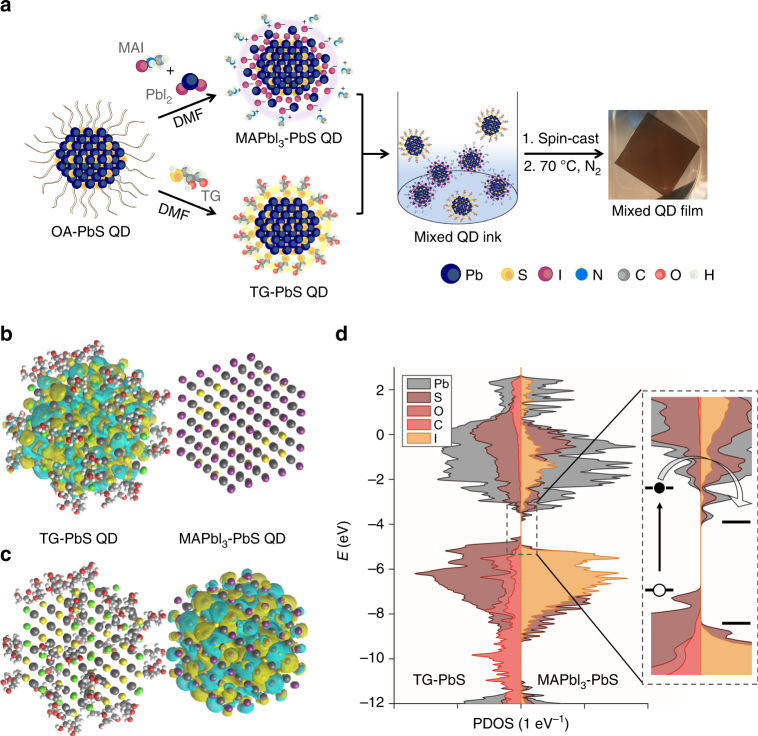
Preparation of mixed-quantum-dot solids using solution ligand exchange. **a** Schematic of the solution ligand exchange process. **b** Calculated HOMO and **c** LUMO states of two coupled dots, showing charge transfer between MAPbI_3_- and TG-capped QDs in the mixed film. **d** Projected density of states demonstrate the offset of the band position between MAPbI_3_- and TG-capped QDs

We began from a recently developed ligand substitution based on a two-phase solution exchange procedure^11^. Concentrated QD inks were directly deposited by spin-casting to yield dense QD films^12^. Donor- and acceptor-type QDs were prepared separately using two sets of solution-phase ligand exchange protocols (Fig. 1a). To form QDs with deeper bands, the oleic acid ligands initially capping the QDs were exchanged with lead halide anions, PbX_3_^−^, to passivate the surface with halogen atoms, yielding electron acceptor (henceforth called A-Type) dots. For QDs with shallower bands, various small organic molecules bearing thiol groups to anchor to the QD surface are used in a separate exchange to form electron donor (D-type) dots. The best performing devices and controls were achieved using a mixture of organohalide perovskite ligands based on methylammonium lead triiodide (MAPbI_3_) for A-type QDs, and thioglycerol (TG) for donor (D-type) dots. Oleic acid and excess unreacted ligands were removed via purification (see Methods). Following the exchange, the hydrophilic functional groups, OH in the case of TG, and MA^+^ and PbI_3_^−^ in the case of the perovskite ligands, render each type of QD dispersible in butylamine, allowing the production of concentrated QD inks. After the exchange process, the QDs maintain their uniform size distribution, with an average diameter of 3.3 ± 0.5 nm (Supplementary Fig. 1). An amorphous layer is observed on the MAPbI_3_-capped QD surface, and this crystallizes to form a thin shell of perovskite under 70 ^o^C annealing used in subsequent processing^13^.

We then prepared mixed-QD inks comprising programmable ratios of D:A types of QDs. The mixed ink was spin-coated in a single step onto substrates coated with ZnO nanoparticles, forming smooth and uniform active layer thin films. Thiol-based ligands are known to move the bandstructure up to shallower energy levels, whereas halide anions deepen the bandstructure^10^. The mixed-QD solid therefore incorporates a nanoscale-distributed band offset that separates electrons and holes into different domains, thus, preventing their subsequent recombination. Both density functional theory (DFT) calculations (Fig. 1b–d; Supplementary Table 1) and ultraviolet photoelectron spectra (Supplementary Fig. 2 and Table 2) confirm this staggered offset in the case of a 3 nm MAPbI_3_-capped QD adjacent to a TG-capped QD, exhibiting a band alignment that favors charge separation into separate carrier pathways at D/A interfaces.

### Quantum dot surface characterization

Various types of QDs, including PbS and PbSe, are known to have surfaces prone to atomic rearrangement, as well labile ligand–surface bonds. Exchange among ligands for A-type and D-type QDs during mixing of the two classes would result in mixed coverage, and negate the benefit of altering the levels of the bands. We define chemical orthogonality as a property of the QD mixture wherein, in the two distinct classes of QDs, each class of QD maintains its surface ligands throughout mixing in solution and in a film.

**Fig. 2:**
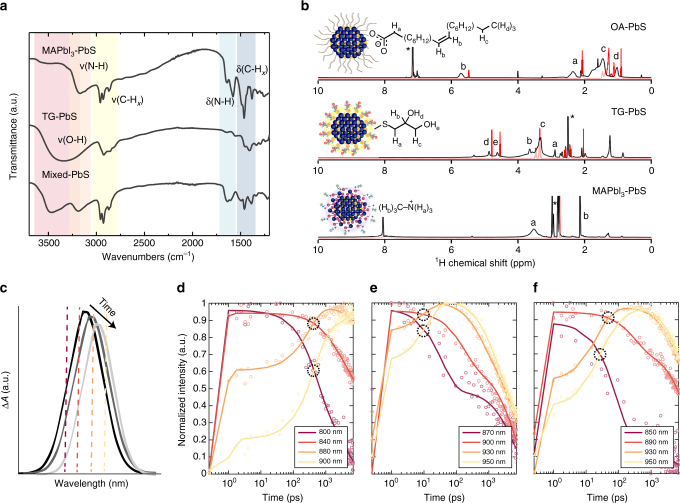
Characterizations of ligand attachment and transient absorption photophysics. **a** Fourier transform infrared (FT-IR) spectra of PbS QDs passivated by (i) MAPbI_3_ (ii) TG, and (iii) two types of ligands (mixed-QD sample). **b** ^1^H NMR spectra of quantum dot ligands in solution (red) and bound to PbS quantum dot surface (black). (i) Oleic acid and lead oleate-capped QDs in C_6_D_6_ benzene. (ii) TG and TG-capped QDs in d_6_-DMSO. (iii) MAI + PbI_2_ solution sample and MAI + PbI_2_-capped QDs in d_7_-DMF. For (ii) and (iii), signals between 0 and 2 ppm are from residual oleate bound to the QDs after the ligand exchange. Peaks from trace nondeuterated solvents are marked by asterisks. **c** Schematic representation of spectral diffusion. Dashed lines indicate the position of time traces for panels **d**–**f** relative to the peak maximum of the exciton bleach. **d**–**f** TA spectral time traces of bleaching signals for **d** TG-capped QDs, **e** MAPbI_3_-capped QDs, and **f** mixed-QD films. We track spectral diffusion by the point of crossover between the decay of high-energy wavelengths and the rise of lower energy wavelengths, encircled in black

To investigate whether we had indeed achieved QD chemical orthogonality, we used Fourier-transform infrared (FT-IR) spectroscopy (Fig. 2a) to characterize the exchanges and the ligand exclusivity of the resultant materials. The absence of a carboxylic group C = O stretch (at around 1750 cm^−1^) from each exchanged QD sample indicates a complete ligand exchange and oleic acid removal. For MAPbI_3_-capped QDs, stretching and bending signals for C–H_*x*_ (2850–2950 and 1380–1460 cm^−1^, respectively) and N–H_*x*_ (3000–3300 and 1550–1700 cm^−1^, respectively) confirm the presence of the MA^+^ cation. The TG-capped QDs, in contrast, show no narrowband N–H_*x*_ signals, but instead reveal a broad and strong peak 3000–3600 cm^−1^, assigned to the O–H stretch of the terminal hydroxyl groups. After we mix the two classes of QDs, the N–H_*x*_ and O–H signals are retained. The O–H stretching signal shifts to a higher wavenumber and exhibits now a sharper lineshape. This is consistent with the previously reported disruption of intermolecular/intramolecular hydrogen bonds among surface TG molecules^14^. We conclude that, when the two classes of QDs are mixed, intramolecular hydrogen bonds are disrupted, and the hydroxyl groups on the TG molecule interact with perovskite functional groups. These chemical bonding features are retained after 70 °C annealing (Supplementary Figs. 3 and 4), indicating that each class of ligand remains strongly bound to each class of QD.

We further studied the two types of terminal functional groups using ^1^H nuclear magnetic resonance (NMR) spectroscopy. Comparisons among the spectra of functionalized QDs (Fig. 2b; Supplementary Figs. 9 and 11) vs. free ligands (Supplementary Figs. 5–8) indicate that each type of ligand binds to the QD surface following solution exchange. This is evidenced by spectral shifting and broadening, the latter arising from slower tumbling of the molecules^15–17^ and an inhomogeneous magnetic QD surface^15, 18^. Crucially, 1D and 2D (NOESY) ^1^H NMR of a 1:1:1 ligand mixture (a mixture of TG, MAI, and PbI_2_ in DMF) reveals no cross-reactions among the various input ligands in solution. Instead, intermolecular hydrogen bonds are formed among the hydroxyl groups of TG ligands and the iodide anion of the PbI_2_ + MAI mixture (Supplementary Figs. 9 and 10)^19^.

These hydrogen bonds account for the homogeneous dispersion of D-type and A-type dots in the QD film. Hydroxyl groups of TG ligands are not reactive enough to bind to the QD surface, and they therefore remain protonated, maintaining their ability to act as hydrogen bond donors. The TG is not reactive with the MAPbI_3_-capped QDs because the robust Pb–S bond prevents ligand dissociation^20^. In comparison, for the MAPbI_3_-capped QDs, it is known that stable ionic shells of [PbI_3_]^−^ form a Stern layer on the surface of the dot, while cationic species such as MAI^+^ and [PbI]^+^ form a diffuse layer^19^. This electrical double-layer maintains charge neutrality on the surface, therefore stabilizing the QD in solution. Due to the strong surface stabilization of both ligands, it is rare under ambient conditions for chemical reactions to occur in solution between the acceptor and donor QDs. These findings suggest that the mixtures of the QDs explored herein should be expected to preserve their distinct optoelectronic properties when incorporated into devices. It also illustrates two distinct colloidal stabilization strategies in a polar solvent, i.e., solubility due to hydrophilic functional groups for small organic molecules in the case of D-type QDs, and charged surfaces in the case of A-type QDs, meaning this strategy is applicable to other donor/acceptor type surface molecules.

### Interdot carrier transfer and exciton dissociation studies

We further tested this picture of surface ligands retained on each type of dot throughout mixing and annealing by investigating the photophysical properties of single-class QD vs. mixed-QD materials. In the mixed-QD BHJ solid, the rate of exciton dissociation is expected to be increased due to type II band alignment throughout the film, and interdot carrier transfer rates are expected to be intermediate between those of pure A-type and pure D-type films. To investigate further how the mixture of D- and A-type QDs influences excited state dynamics in the mixed-QD solid, we performed ultrafast transient absorption (TA) spectroscopy (see Supplementary Note 1 for detailed explanation of TA spectroscopy). Following photoexcitation, the carriers diffuse throughout the film from higher to lower energy sites due to the inhomogeneity in the QD solid. This will cause a time-dependent redshifting and narrowing of the exciton signal in TA spectra, schematically shown in Fig. 2c (data for QD films shown in Supplementary Fig. 12). The rate of this process, called spectral diffusion, is directly related to the mobility of the carriers as they navigate the energy landscape, and is indicated by the more rapid decay of the signal at higher energies, mirrored by a rise of the signal at lower energies^21^. Such time traces are plotted for solids of TG-capped, MAPbI_3_-capped, and mixed-QD films in Fig. 2d and f, respectively. Relative rates of spectral diffusion are estimated by the temporal crossing of these high- and low-energy time traces for the different QD solids, and are found to be slowest in TG-capped QDs (crossover at 345 ps), fastest in MAPbI_3_-capped QDs (7.5 ps), and intermediate in the mixed-QD solid (12 and 40 ps). Spectral diffusion in the mixed-QDs comes closer to the timescale of the MAPbI_3_-capped QDs, consistent with the preponderance (2:1 ratio) of MAPbI_3_- to TG-capped QDs. Higher-power TA data reconfirm the intermediate dynamics of the mixed-QD film based on comparing the rates of diffusion-assisted Auger recombination (Supplementary Fig. 13 and Supplementary Table 3). We conclude that the carrier mobility is highest for MAPbI_3_-capped QDs, lowest for TG-capped QDs, and intermediate between these two extremes for the mixed-QD film. Low mobility in the TG-capped QDs contributes to their poor device performance compared to pure MAPbI_3_-capped QDs. Additional analysis of TA spectral features is found in Supplementary Note 1.

Importantly, despite the lower mobility in mixed-QD films, time-resolved PL spectroscopy indicates faster exciton dissociation in mixed-QDs compared to MAPbI_3_-capped and TG-capped QD films (Supplementary Fig. 14). While TA signal measures carriers, time-resolved photoluminescence (TRPL) measures excitons and, therefore, is sensitive to exciton dissociation into free carriers, manifested as a rapid decay of the PL signal. This is a strong indication for the formation of a BHJ, where the staggered band alignment between D- and A-type QDs throughout the film favors charge separation of carriers into different phases.

**Fig. 3:**
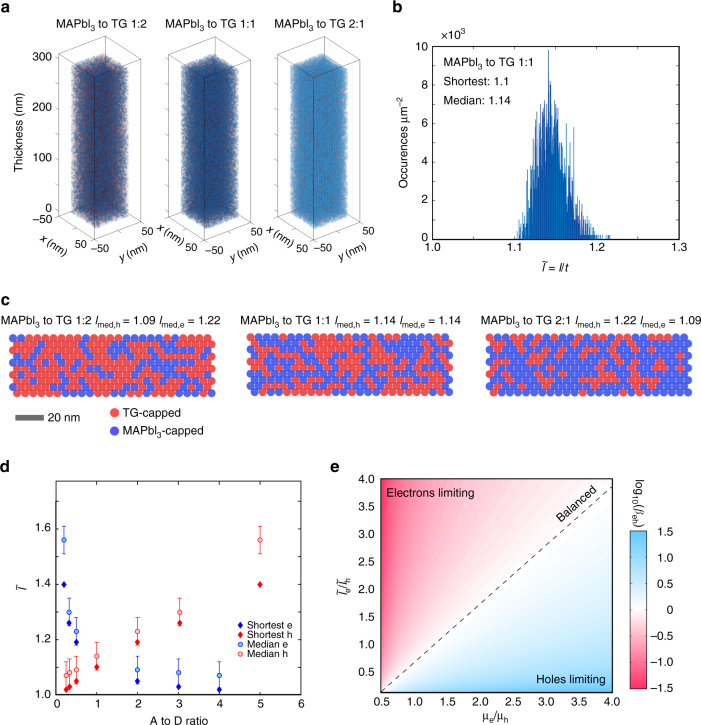
Morphology of the mixed-quantum-dot structure for balanced carrier extraction. **a** Cross-section of a mixed film with different QD donor/acceptor ratios. Blue and red spheres represent acceptor and donor QDs, respectively. **b** Distribution of path lengths for a 1:1 A- to D-type QD ratio. For this configuration, the shortest path (normalized to thickness) is 1.1, and the median length of 1.14. **c** Slices of mixed CQD solids with different D/A ratios representing the dominant presence of each type of QD. **d** Normalized path length as a function of A- to D-type QD ratio within a mixed QD film. Error bars represent the standard deviation of the 10 shortest different path lengths for each A/D configuration. **e** Balanced extraction (*β* = 1) requires a compromise between electron and hole mobilities and their respective path-lengths. A maximum power conversion efficiency is expected when the carrier extraction is balanced (Supplementary Fig. 14)

The appreciable difference between the mobilities of D-type and A-type QDs suggested the following possible solution: vary the D-/A-ratio in the film to modulate the overall transport of charge in the BHJ (i.e., mixed QD) active layer. We began by evaluating how the assembly of mixed-QD inks into solid films may impact the extraction of photogenerated carriers (Fig. 3). The ratio of D-type to A-type QDs will determine the abundance of percolation pathways for holes vs. electrons (Fig. 3a). A statistical analysis of the network morphology sheds light on the most likely pathways for efficient carrier extraction (Fig. 3b, c). In a 1:1 donor:acceptor film, the shortest path (*l*) for each carrier class has a length 1.1 *t*, where *t* is the film thickness. The normalized path length ($\tilde l = l/t$) showcases a narrow distribution with a median of 1.14. When we vary the A/D ratio (Fig. 3d) across the 0.3–3 range, the length imbalance remains below 20%, ensuring substantially similar electron vs. hole collection distances. The carrier extraction time must also take the mobility into account: the condition for balanced extraction occurs when the electron and hole transit times coincide (Fig. 3e):

1 $$\beta = \frac{{{t_{\rm{e}}}}}{{{t_{\rm{h}}}}} = \frac{{{l_{\rm{e}}}}}{{{l_{\rm{h}}}}}\frac{{{\mu _{\rm{h}}}}}{{{\mu _{\rm{e}}}}}$$

In the context of asymmetric transport in D- and A-type QD domains, control over the donor:acceptor ratio may provide a means to recover balanced transport. A 4-power-points improvement in PCE can be accomplished as *β* approaches unity based on the results of SCAPS simulations^22^ (Supplementary Fig. 15). The importance of balanced charge extraction for achieving high-efficiency photovoltaics has been highlighted in this architecture, which is further supported by experimental results (Supplementary Fig. 16).

**Fig. 4:**
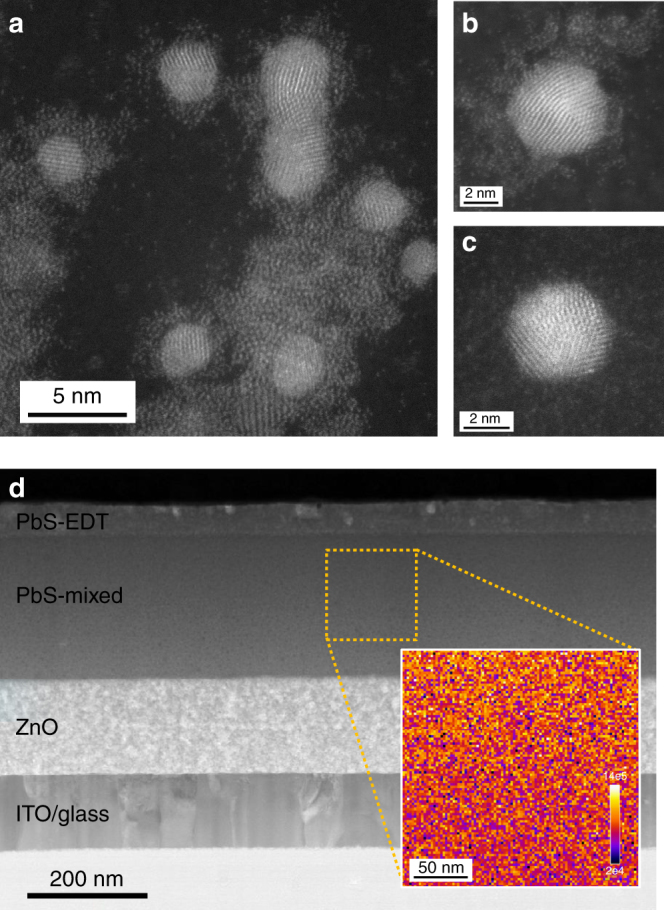
Electron microscopy analysis of mixed-quantum-dot solid and device. **a** STEM-HAADF image of freshly drop-cast mixed-QD ink on an ultrathin graphite-based TEM grid (sample was not annealed). QDs with amorphous MAPbI_3_ shell (**b**) and without surface shelling (**c**). **d** Cross-section STEM image of FIB-processed mixed QD-based photovoltaic device. Inset: EELS mapping of iodine in the highlighted region (200 nm by 200 nm) indicating the homogenous dispersion of MAPbI_3_-passivated QD within the mixed QD active layer (major edge of iodine M_4,5_ signal starting at around 625 eV, signal intensity unit: a.u., Supplementary Fig. 19)

We therefore pursued systematic optimization of the D/A stoichiometry to enable improved mixed-QD-based photovoltaics. First, we investigate the nature of the mixed-QD solid. High-angle annular dark-field scanning transmission electron microscopy (HAADF-STEM, Fig. 4a) imaging of a drop-cast mixed-QD sample (D-type/A-type mass ratio = 1:1, sample not annealed) reveals crystalline QD cores surrounded by amorphous shells (Fig. 4b) that resemble closely the analogous images of the pure phase of MAPbI_3_ dots (Supplementary Fig. 1). It also includes QDs that lack amorphous shelling (Fig. 4c) and agree with the pure TG dot images. We conclude that the two independent classes of QDs are maintained in the mixture and in solids made from it. Electron energy loss spectroscopy (EELS) imaging and bright-field TEM and energy-dispersive X-ray (EDX) microanalysis of focused ion beam (FIB) cross-sectioned samples (Fig. 4d and Supplementary Figs. 17–19) indicate no signs of phase segregation into multi-dot domains throughout the thickness of the active layer.

### Optoelectronic characterization and device performance

**Fig. 5:**
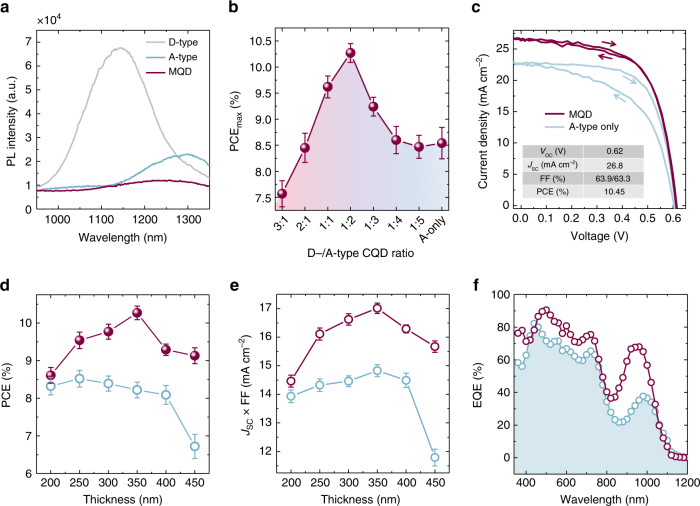
Optoelectronic behaviors of mixed-quantum-dot solids and device performance. **a** Photoluminescence spectra of TG-, MAPbI_3_-capped, and mixed QD films confirming the quenching effect on mixed QD sample because of efficient charge separation between donor/acceptor QDs. **b** Power conversion efficiency (PCE) values of mixed QD devices with different mass ratios of D- (TG-capped) and A-type (MAPbI_3_-capped) QDs. **c** Current−voltage (*J*−*V*) characteristics under simulated AM 1.5 illumination for BHJ device and control A-type only (MAPbI_3_-capped) PbS device. **d** Thickness-dependent PCE and **e** thickness dependent of BHJ and control perovskite-passivated QD devices. **f** EQE spectra of champion-mixed QD device and perovskite-passivated QD control sample. Error bars in **b**, **d**, and **e** represent the standard deviation of several devices

The effect of the band offset between D/A QDs, which contributes to electron-hole pair dissociation, can be characterized using photophysical studies. When comparing the PL brightness of the mixed QD film to its separate precursor components, the QY is 34× and 12× lower than that of individual TG- and MAPbI_3_-capped samples, respectively (Fig. 5a). We then constructed planar structural devices that allowed us to pursue increased performance for a QD ink, and also connect our model-based studies of D/A ratio with actual performance. The active mixed-QD layer was sandwiched between a ZnO nanocrystal layer (the electron transport layer) and 1,2-ethanedithiol (EDT)-passivated QDs (the hole transport layer) for efficient charge separation. The PCE of the best mixtures benefits appreciably from the incorporation of an optimal concentration of TG-capped QDs (Fig. 5b). An (uncertified) maximum PCE value of 10.45% (average value: 10.26 ± 0.17%) was obtained with a 2:1 MAPbI_3_-QD to TG-QD mass ratio. This is a 23% (relative) increase compared to the control perovskite-passivated QD device and 5× higher than that observed from the pure TG-capped QD-based device (Supplementary Fig. 20). Figure 5c provides a detailed comparison of the record and control devices in current density–voltage (*J*−*V*) characteristics. The marked advance in performance for the mixed-QD device originates mainly from the significant improvement in short-circuit current density (*J*_sc_ reaches an impressive 26.8 mA cm^−2^) due to reduced recombination losses through traps even when traps are present.

The unencapsulated device retains 80% PCE after 10 days of storage in air (Supplementary Fig. 21). The instability of the BHJ devices may originate from the degradation of the TG-capped PbS QDs. X-ray photoelectron spectra of TG-passivated QD films confirm the detachment of TG ligands and the excessive surface oxidation when the sample is exposed to air (Supplementary Fig. 22). This is consistent with previous reports that monothiol-ligand-capped PbS QDs are prone to oxidation by air and moisture^23^.

To investigate the impact of mixing on device performance, we performed control studies on bilayer devices comprised of a pure A layer atop a pure D layer (Supplementary Fig. 23); this led to no improvement in either *J*_sc_ or PCE over the best of {pure A, pure D} devices. This confirms the need to form the nanoscale mixture to overcome the short carrier diffusion length in MAPbI_3_-capped QD films. Another crucial control was the construction of devices based on dots formed using a mixture of starting ligands with optimized QD thickness (Supplementary Figs. 24–26). FT-IR experiments reveal that each dot is co-passivated by TG and MAPbI_3_ ligands (Supplementary Fig. 27). The characteristic stretching and bending peaks match that of the mixed-QD film due to the same type of ligand chemical interactions. Each of these mixed-ligand devices exhibit PCEs below 9% regardless of the ratio between the two types of ligands (i.e., TG and MAPbI_3_). External quantum efficiency (EQE) spectra of the mixed-QD devices surpass those of control devices across the entire spectrum, indicating the extraction length is not improved by increasing the thickness of the active layer in the co-treated devices. This is also demonstrated in thickness-dependent studies of the ligand co-passivated devices. These results further confirm the necessity for a hetero-offset, allowed by orthogonal colloids, and not just co-passivation.

Thickness-dependence studies on mixed QD and control MAPbI_3_-passivated devices allowed us to further characterize the impact of the mixed-QD solid on effective transport lengths. As the active layer thickness is increased from 200 to 350 nm, the mixed-QD device improves consistently in short circuit current density (*J*_sc_) and fill factor (FF). In contrast, the PCE for pure-phase controls decreases systematically when the absorbing layer is made thicker (Fig. 5d, e). The EQE of the mixed-QD device surpasses that of the single-QD ink control across the entire spectrum, again attesting to the benefits of an increased extraction length in the mixed material system (Fig. 5f).

## Discussion

We report herein a mixed-QD ink that enhances charge carrier extraction in QD solar light harvesting layers. The approach relies on forming a mixture of donor and acceptor QDs that each maintain their distinct chemical character when mixed in the solution phase and when formed into a final film. The electron affinity of the QDs is controlled by the application of surface ligands in solution exchanges completed prior to the formation of the colloidal mixture. These ligands are bound tightly to the surface, allowing the intimate mixture of the two dot classes to be preserved even in the solid state, leading to electrons and holes being separated into different domains, suppressing their subsequent recombination. Photocarriers can then migrate efficiently through their respective donor/acceptor transport media when the D:A stoichiometry is suitably optimized. These joint benefits, which arise from surface chemical control and materials processing, ultimately allow us to construct thick solution-processed solar cells that absorb more light and generate more current without a compromise to *V*_oc_ and FF. Further advances in performance may be sought by (i) improving the optoelectronic properties and the stability of the D-type QDs and (ii) achieving overall charge balanced transport throughout the device. We propose—considering rapid progress in QD surface passivation and interface engineering^24, 25^—that future generations of QD devices can benefit from the engineering of balanced charge extraction enabled by this mixed-QD strategy explored herein.

## Methods

### Materials

All chemicals used are commercially available from Sigma-Aldrich (or otherwise specified) and were used without any additional purification steps: lead (II) oxide (99.99%, from Alfa Aesar), cadmium chloride (99.99%), bis(trimethylsilyl)sulfide (synthesis grade) oleic acid (tech. 90%), 1-octadecene (ODE, ≥95%), oleylamine (≥98%), dimethylformamide (DMF, 99%), octane (anhydrous, >99%), butylamine (BA, 99.5%), lead (II) iodide (from Alfa Aesar, 99.999%, ultra dry), methylammonium iodide (MAI, from Dyesol Inc., 99.9%), thioglycerol (TG, 99%), 1,2-ethanedithiol (EDT, 99%) toluene anhydrous, methanol anhydrous, acetone, distilled in glass (Caledon).

### PbS QD synthesis and perovskite ligand exchange

The synthesis of oleic acid-capped PbS QDs follows published methods^26^. For methylammonium lead triiodide (MAPbI_3_) perovskite ligand exchange, 5 ml of purified oleic acid-capped QD octane solution (20 mg ml^−1^) was mixed in a vial with 5 ml of dimethylformamide (DMF) solvent containing equimolar amounts of MAI and PbI_2_ (0.3 mol l^−1^). The solution was mixed vigorously at room temperature for about 10 min to ensure complete ligand exchange; a successful exchange results in a dense, black solution in the DMF layer. The octane layer was decanted and an additional 5 ml of octane was added and vortexed with DMF solution to wash the residual oleic ligands. This purification process was repeated twice. Next, the QD DMF solution was transferred into two test tubes (~2.5 ml each). Approximately 1.5 ml of toluene was slowly added into each tube to precipitate the QDs. This results in a translucent, dark brown solution. The precipitate was isolated by centrifugation at 6000 rpm for 2 min and the supernatant was decanted. The precipitate was dried under vacuum at room temperature for 20 min and finally redispersed by adding the predesigned amount of dry butylamine to form a concentrated ink (e.g., 188 mg ml^−1^ for the preparation a QD film with a thickness of around 300 nm).

### Solution phase thioglycerol ligand exchange

A volume of 50 μl of TG was added into 5 ml of DMF to form a transparent solution (0.114 mol l^−1^). A volume of 5 ml of purified oleic acid-capped QD octane solution (20 mg ml^−1^) was then added and mixed vigorously at room temperature for about 2 min. After ligand exchange, QDs were transferred to the DMF solution phase. The octane supernatant was decanted and an additional ~5 ml of octane was added and vortexed with DMF solution to remove the residual oleic ligands. This purification process was repeated twice. Next, the QD DMF solution was transferred into two test tubes (~2.5 ml each). Approximately 1.5 ml of toluene was added into each tube, resulting in a dark brown and non-transparent solution. The precipitate was isolated by centrifugation at 6000 rpm for 2 min and the clear supernatant was decanted. The precipitate was finally dried under vacuum at room temperature for 20 min then stored inside a nitrogen-filled glovebox for further use.

### Solar cell fabrication

The solar cells were prepared on a pre-patterned ITO substrate (2.5 cm × 2.5 cm). ZnO nanoparticles were prepared following published methods^27^. Two layers of ZnO nanoparticles were deposited on the substrate by spin-coating at 3000 rpm for 10 s. A volume of 40 μl of concentrated and mixed QD ink was then transferred by a micropipette and deposited onto a piece of ZnO nanoparticle/ITO substrate by spin-coating at 3000 rpm for 30 s, forming a thick QD film that was further annealed at 70 °C for 10 min under a nitrogen atmosphere. Two layers of EDT ligand-exchanged QDs were deposited on top of the QD film by spin-casting following reported method^27, 28^. For the top electrode, 120 nm Au was thermally deposited on the PbS film to complete the device. Each ITO substrate was patterned to yield eight devices, each with an area of 7.1 mm^2^.

### PL and absorption measurement

Photoluminescence measurements were done with a Horiba Fluorolog Time Correlated Single Photon Counting system equipped with UV/Vis/NIR photomultiplier tube detectors, dual grating spectrometers, and a monochromatized xenon lamp excitation source. Optical absorption measurements were carried out in a Lambda 950 UV-Vis-IR spectrophotometer.

### FTIR measurement

The transmission mode FTIR samples were done by drop casting the quantum-dot inks on a glass substrate. The Thermo Scientific Nicolet iS50 ATR-FTIR was used to obtain the FTIR spectra. Spectra were obtained using 16 scans with a resolution of 4 cm^−1^, and the collection range was between 550 and 4000 cm^−1^.

### ^1^H NMR measurements of QDs

All NMR experiments were carried out on a Bruker Avance III 400 instrument. All samples were scanned 128 times with a 15-s relaxation delay. NMR samples for oleate-capped QDs were prepared by evaporating a solution of 50 mg ml^−1^ of QDs in octane and redispersing them in C_6_D_6_ benzene. Approximately 1 ml of deuterated solvent was used for ~40 mg of QDs for NMR measurements to obtain good signal-to-noise ratio.

For the TG exchanged QDs, the spectrum contains many solvent peaks from DMF and toluene, in addition to the residual solvent peak from d_6_-DMSO. These QDs were prepared without deviating from the TG exchange protocol. Re-dispersing the QDs in DMF and re-precipitating them with toluene, or drying the sample after the initial precipitation with toluene, rendered them insoluble in DMSO. To a 15-ml glass test tube containing ~50 mg of solid freshly precipitated sample, ~1 ml of d_6_-DMSO is added. The test tube is vigorously agitated for 1 min via a Vortexer and then sonicated for 5 min before being transferred to a 5 mm NMR tube. TG binding onto the QD surface via the thiol group is evidenced by the disappearance of the H-S signal (∂ = 2.05 ppm, s, 1 H) and the broadening of all other TG signals. Because of the poor dissolution of the TG-capped dots in DMSO, we attribute very broad peaks (~1 ppm) centered at the QD-bound TG peaks to QDs that have aggregated into larger solids that are suspended in solution.

NMR samples for perovskite-capped QDs were prepared from freshly precipitated QDs directly after the solution ligand exchange. Approximately 40 mg of perovskite-capped QDs are dissolved in ~1 ml of d_7_-DMF to obtain good signal-to-noise ratio.

### X-ray diffraction

X-ray diffraction (XRD) samples were done by spin coating the QD inks on a glass substrate. Measurements were taken by using the Rigaku Miniflex 600 diffractometer with a NaI scintillation counter and using monochromatized Cu-Kα radiation (1.5406 Å). XRD spectra were scanned between 2*θ* ranges of 5–50 °C with an integration of 0.5 s.

### Photoelectron spectroscopic measurements

A layer of the respective CQDs were spin-casted onto an Au substrate before UPS testing. The ultraviolet photoelectron spectra were obtained using the 21.22 eV He I lines from the discharge lamp. X-ray photoelectron analysis was carried out using Thermo Scientific K-Alpha XPS system with an Ar ion gun.

### *J*–*V* characterization

Current–voltage traces were acquired with a Keithley 2400 sourcemeter unit under simulated AM1.5G illumination (Sciencetech class A). The spectral mismatch was calibrated using a reference solar cell (Newport), yielding a correction multiplicative factor of *M* = 0.994. Devices were measured under a continuous flow of nitrogen gas. The aperture was 4.9 mm^2^ for solar cell measurement.

### EQE measurement

EQE spectra were taken by measuring the photocurrent generated after subjecting the cells to monochromatic illumination (400 W Xe lamp passing through a monochromator with appropriate cutoff filters, calibrated with Newport 818-UV and Newport 838-IR photodetectors). The beam was chopped at 220 Hz. The response of the cell was acquired with a Lakeshore pre-amplifier connected in series to a Stanford Research 830 lock-in amplifier at short-circuit conditions (virtual-null).

### Electron microscopy and mapping characterization

All electron microscopy measurements were performed at the Canadian Centre for Electron Microscopy. TEM samples were prepared by drop casting TG-, and MAPbI_3_-capped PbS QD solutions onto ultra-thin carbon 3 mm grids. The loaded grids were allowed to dry under a hot lamp for 5–10 min. Some samples were subject to short plasma cleaning (10–20 s) to reduce the effects of beam contamination inside the electron microscope.

FIB technique was applied for the preparation of solar cell TEM sample using Zeiss NVision 40 FIB-SEM. The area of interest was coated with an ion-beam deposited protection layer of tungsten. The lamella under the area of interest was liberated by FIB milling and attached to a manipulator needle, followed by attachment to a post of a TEM half-grid. The lamella was thinned from both sides using line milling with the 30 kV Ga ion beam and the final probe current down to 40 pA. This was followed by a lower voltage polishing step on both sides at a 7º glancing angle with a 10-kV ion beam, and then again 5 kV.

TEM analysis was performed using an aberration-corrected FEI-Titan Cubed electron microscope, equipped with an Oxford EDX detector and a Gatan EELS spectrometer, and operated at 200 kV for high-resolution imaging and 80 kV for imaging and EDX analysis. A combination of high-angle annular dark-field scanning TEM (HAADF-STEM) imaging and high-resolution TEM (HRTEM) imaging modalities were utilized to image individual nanoparticles and nanoparticle ensembles. In addition, energy-dispersive X-ray (EDX) microanalysis was used to map the atomic species present in the various layers of the device and EELS was used to map the location of iodine in the active layer of the solar cell device.

### Carrier extraction and morphology in the mixed-QD solid

The normalized path length ($\tilde l = l/t$) distribution was modeled as follows: first, a volume of 80 × 80 × 300 nm^3^ was randomly filled with D and A QDs based on a uniform distribution and the D to A ratio. Percolation paths over this volume were calculated following the Dijkstra algorithm, and shortest and median lengths obtained for different ratios. Details on SCAPS optoelectronic simulations can be found in Supplementary Table 4.

### TRPL spectroscopy

The time-resolved photoluminescence spectroscopy measurements were performed using the Horiba Fluorolog Time-Correlated Single Photon Counting (TCSPC) system equipped with UV/Vis/NIR photomultiplier tube detectors, dual grating spectrometers, and a monochromatized xenon lamp excitation source. The film was placed at an incident angle of 30° away from the detector to avoid reflections of the incident beam. A 375 nm laser diode was used as a pulsed excitation source, and the time traces were acquired using the TCSPC near-infrared detector. The time window was set appropriately to ensure a complete decay of the photogenerated carriers.

### Ultrafast TA spectroscopy

Femtosecond laser pulses of a 1030 nm fundamental beam at a 5-kHz repetition rate were produced using a regeneratively amplified Yb:KGW laser (PHAROS, Light Conversion). Part of the fundamental beam was used to pump an optical parametric amplifier (ORPHEUS, Light Conversion) to serve as a narrowband pump, while the other part was focused into a sapphire crystal to generate a white-light supercontinuum probe (900–1000 nm window with various optical filters). Both the pump and probe pulses were directed into a commercial transient absorption spectrometer (Helios, Ultrafast). Delaying the probe pulse relative to the pump provides a time window of up to 8 ns, and the time resolution of these experiments was ∼300 fs (estimated by the rise time of signal amplitudes in transient absorption spectra). TA measurements on QD films were performed at low powers to extract band tailing dynamics and at high powers to extract Auger lifetimes and dynamics. QD films were pumped at slightly shorter wavelengths than their bleach maxima to avoid pump scatter. TG-capped QD films were pumped at 820 nm (bleach maximum ~840 nm), MAPbI_3_-capped QD films were pumped at 890 nm (bleach maximum ~910 nm) and BHJ-QD films were pumped at 870 nm (bleach maximum ~890 nm).

### Data availability

All data are available upon request.

## Electronic supplementary material

Supplementary Information
