# Supplementary material for: Mixed-quantum-dot solar cells
Source: Nat Commun. 2017 Nov 6;8:1325. doi: 10.1038/s41467-017-01362-1 (PMC5673898; doi:10.1038/s41467-017-01362-1)
Supplement: Supplementary file 1 — Supplementary Information [file 41467_2017_1362_MOESM1_ESM.pdf]

SUPPLEMENTARY FIGURES:

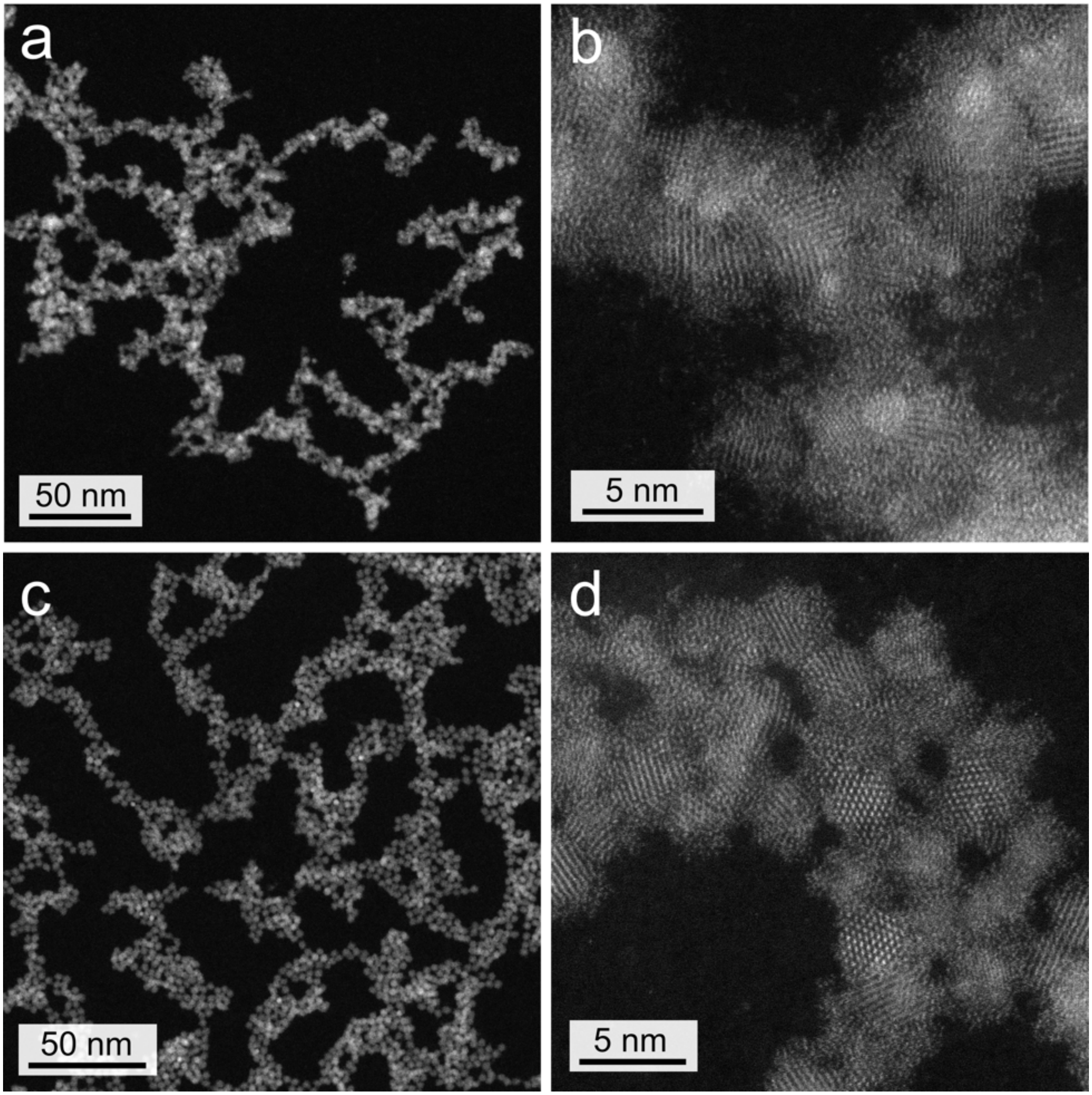

Supplementary Figure 1 | STEM-HAADF images of PbS QDs with different surface ligand passivation. **(a)** Low and **(b)** high resolution of amorphous $MAPbI_3$-passivated QDs. **(c)** Low and **(d)** high resolution of TG-passivated QDs.

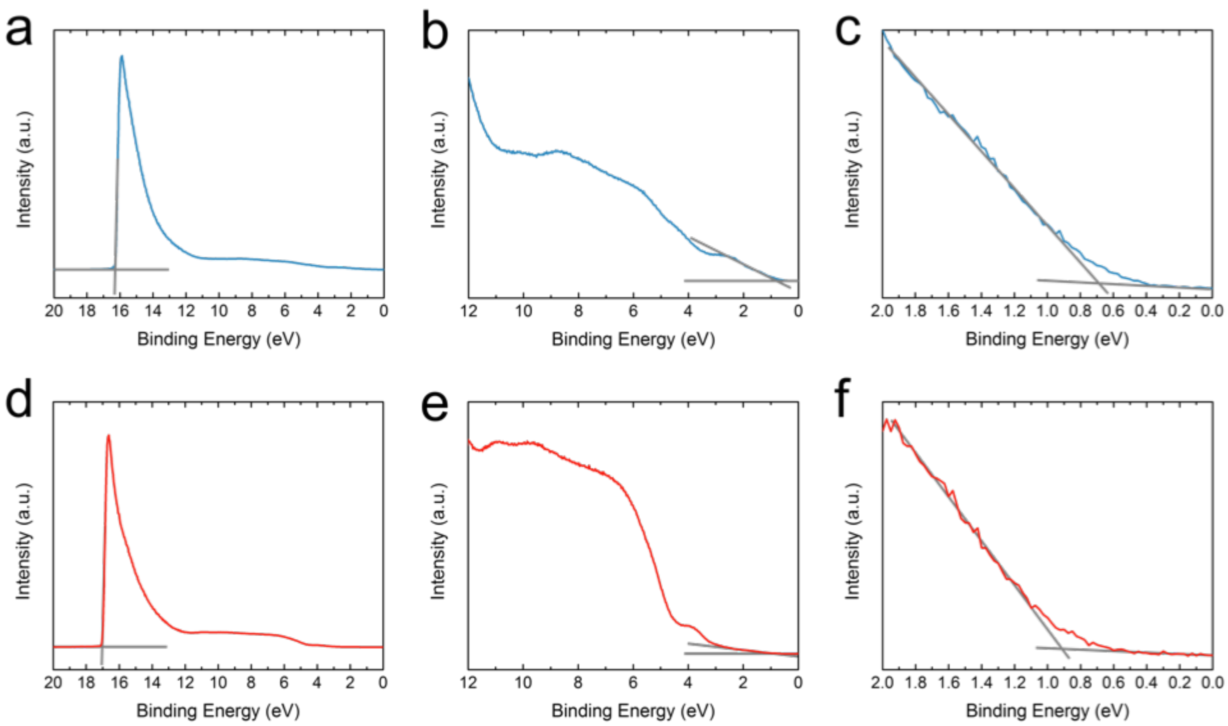

Supplementary Figure 2 | Ultraviolet photoelectron spectra (UPS) of $MAPbI_3$- (blue) and TG-capped (red) PbS QDs (a, d) Complete UPS of ~200 nm thick ligand-passivated PbS QD film on gold. **(b, e)** zoom-in spectra (binding energy: 0-12 eV) of the same samples. **(c, f)** magnified views of the low-binding-energy cutoff regions. The band energies are determined from the intersection of a linear extrapolation to the baseline (both highlighted in gray).

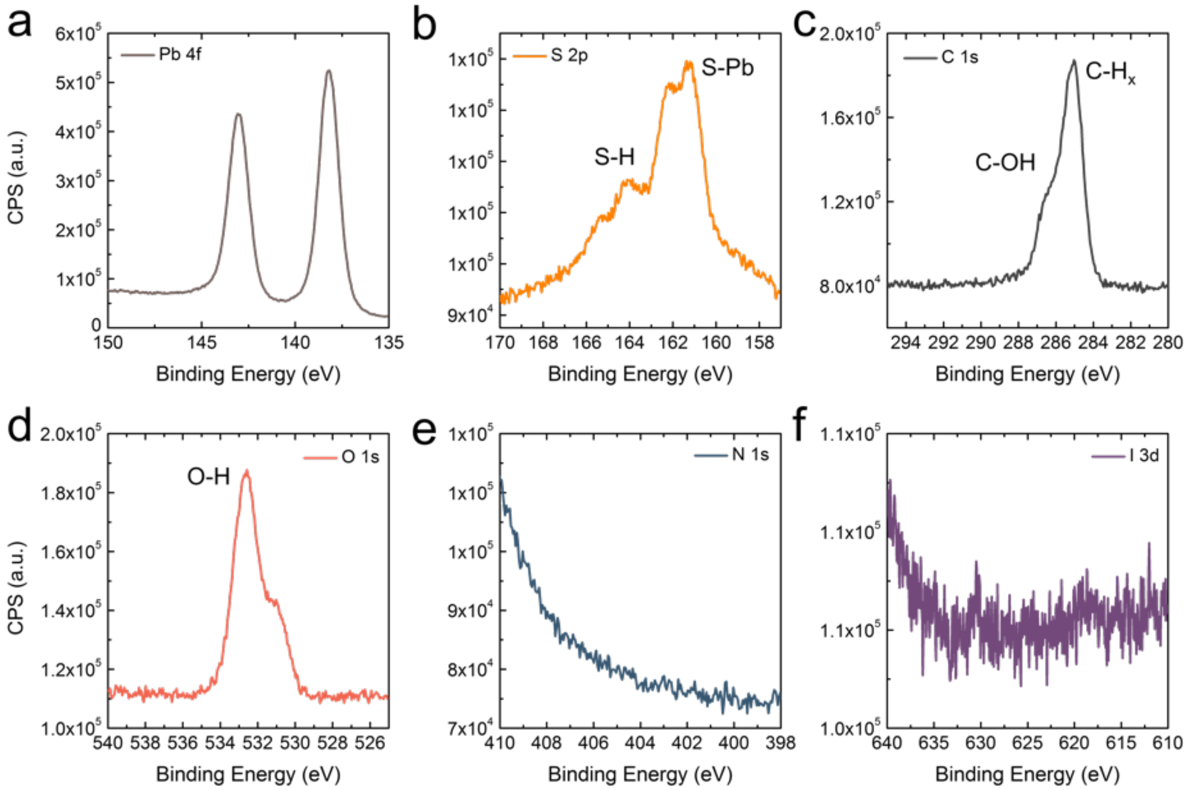

Supplementary Figure 3 | High resolution XPS results of different elemental regions of annealed TG-capped PbS QD film: **(a)** lead 4d; **(b)** sulfur 2p; **(c)** carbon 1s; **(d)** oxygen 1s; **(e)** nitrogen 1s; **(f)** iodine 3d. The thiol signals at ~164 eV in **Figure b** confirms the TG attachment on PbS surface. The absence of nitrogen signal in **Figure e** confirms the complete removal of butylamine after annealing step. No iodine feature is observed from the TG-passivated PbS film (**Figure f**).

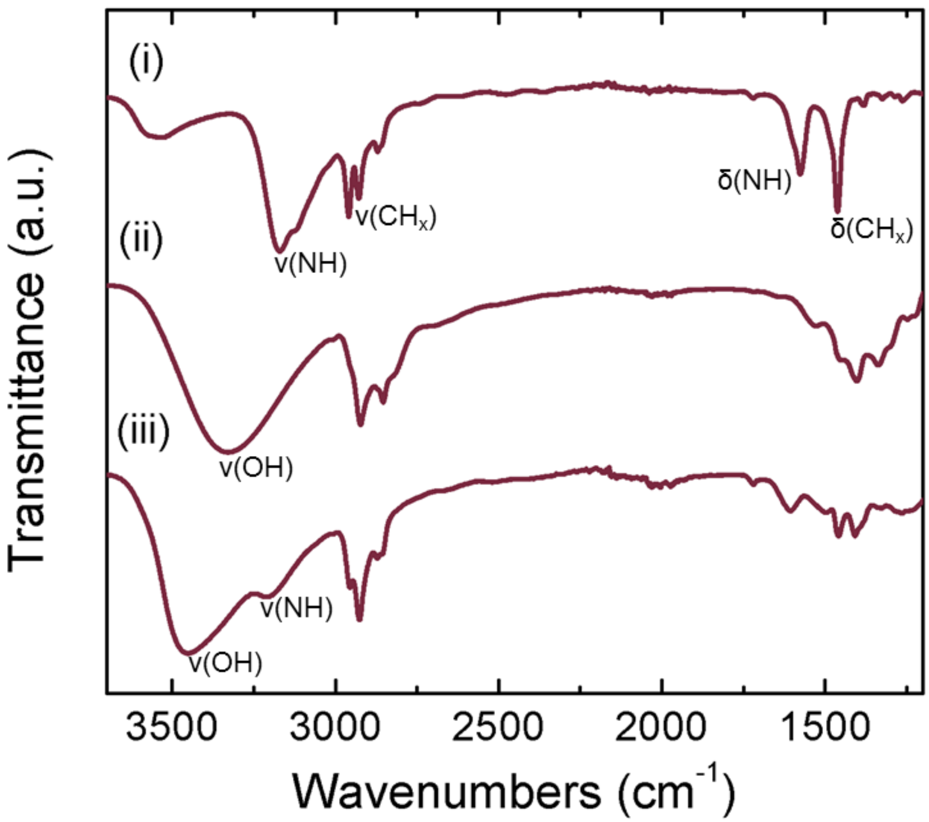

Supplementary Figure 4 | FT-IR spectra of QD films after annealing: (i) $MAPbI_3$-capped QDs (ii) TG-capped QDs and (iii) mixed-QD films. No change in chemical-bonding signal positions is found from these spectra when compared with those from spectra of samples before annealing (**Figure 2a**).

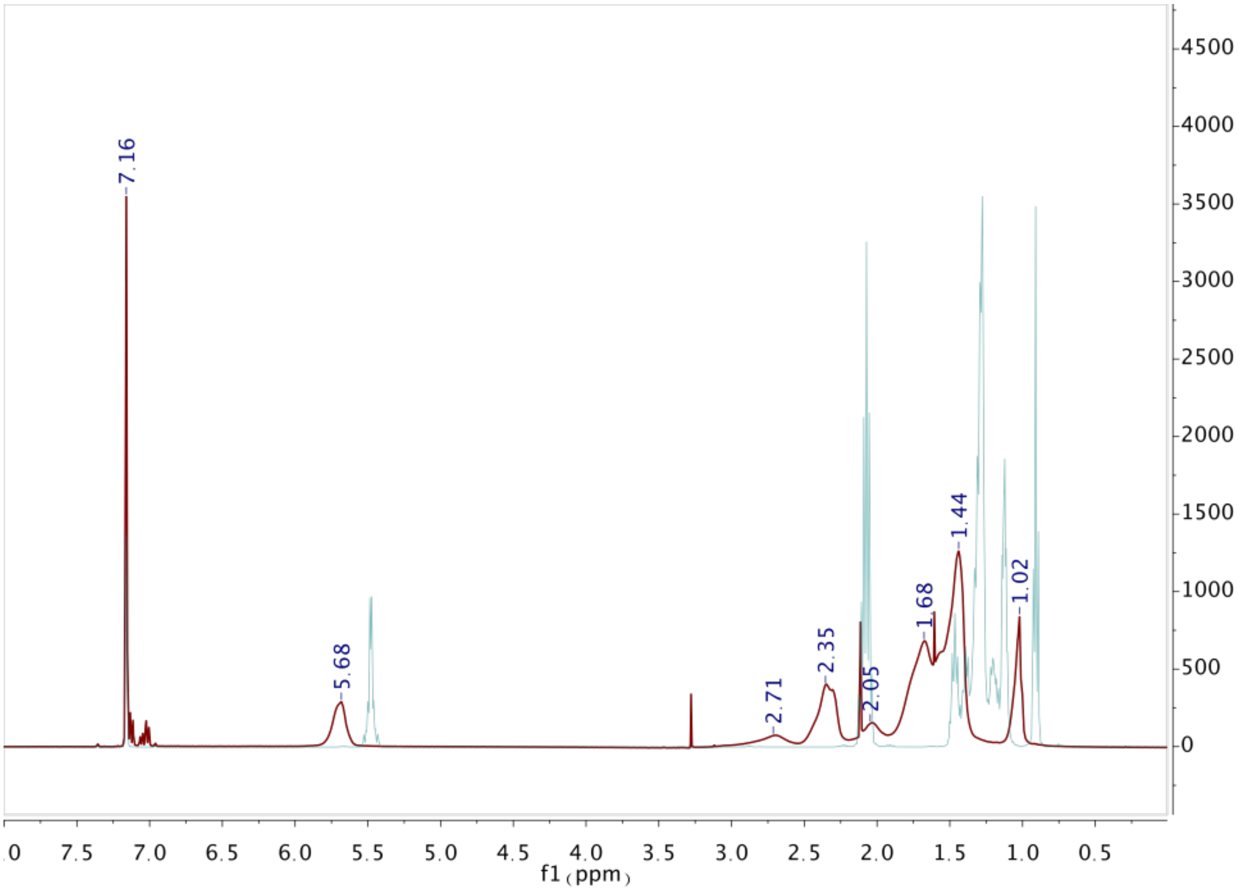

Supplementary Figure 5 | ^{1}H NMR for oleic acid molecule (light blue peaks) and oleate-capped QDs (red peaks) in C_6D_6 benzene. Spectra referenced to residual nondeuterated benzene peak at 7.16 ppm. Peaks belonging to bound oleate are CH_3 (∂ = 1.02 ppm), CH_2 (∂ = 1.44 – 2.71 ppm), and HC=CH (∂ = 5.68 ppm).

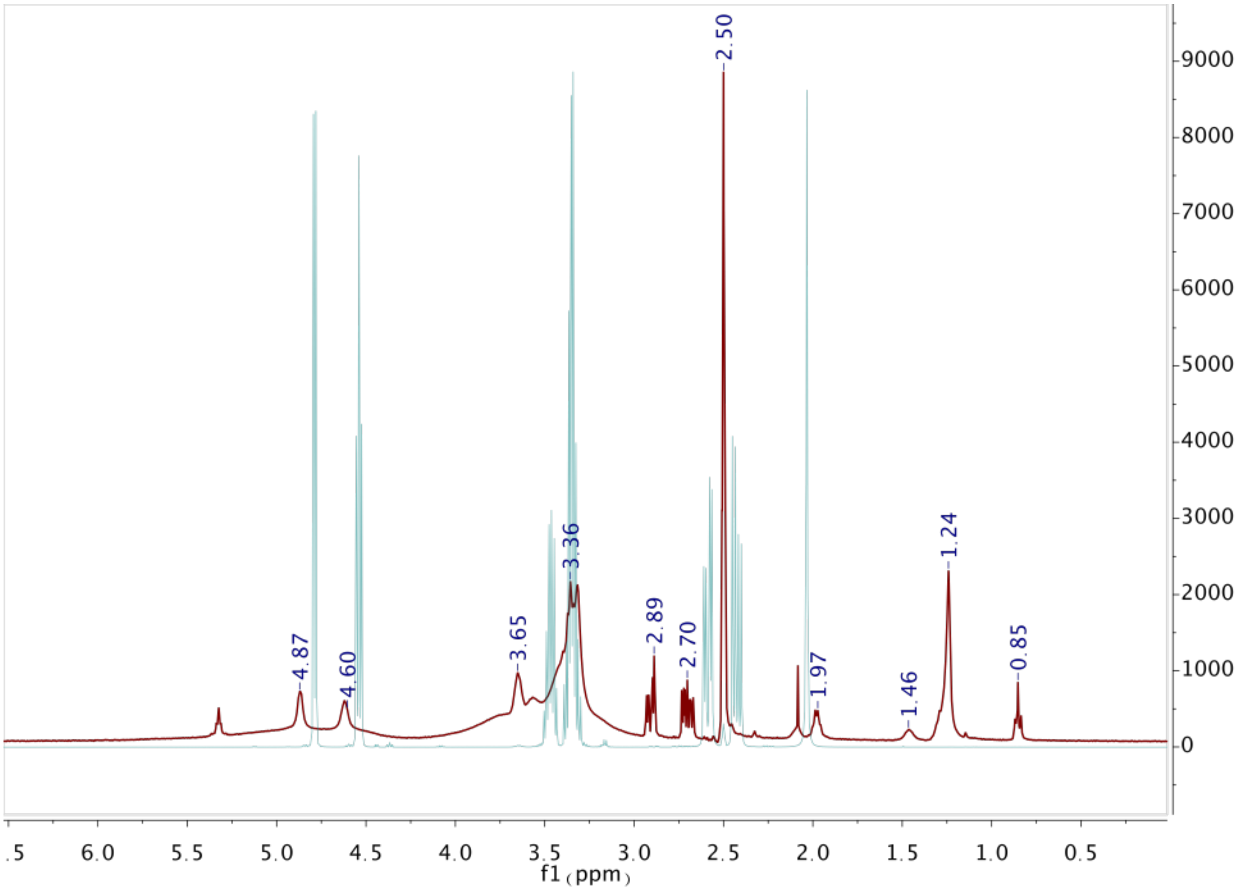

Supplementary Figure 6 | ^{1}H NMR for TG molecule (light blue peaks) and TG-capped QDs (red peaks) in d_6-DMSO. Spectra referenced to residual nondeuterated dimethyl sulfoxide (DMSO) peak at 2.50 ppm. We attribute much broader signals in TG-capped dots not solely due to lower T2 lifetimes, tumbling, or magnetic inhomogeneity but also to small solid aggregates that form due to the low colloidal stability of these QDs in DMSO. Peaks between ∂ = 0.85 - 1.97 ppm and at ~5.4 ppm are from residual oleic acid / bound oleate. Peaks belonging to bound TG are C-H (∂ = 2.70, 2.89, 3.36, 3.65 ppm) and O-H (∂ = 4.60 and 4.87 ppm).

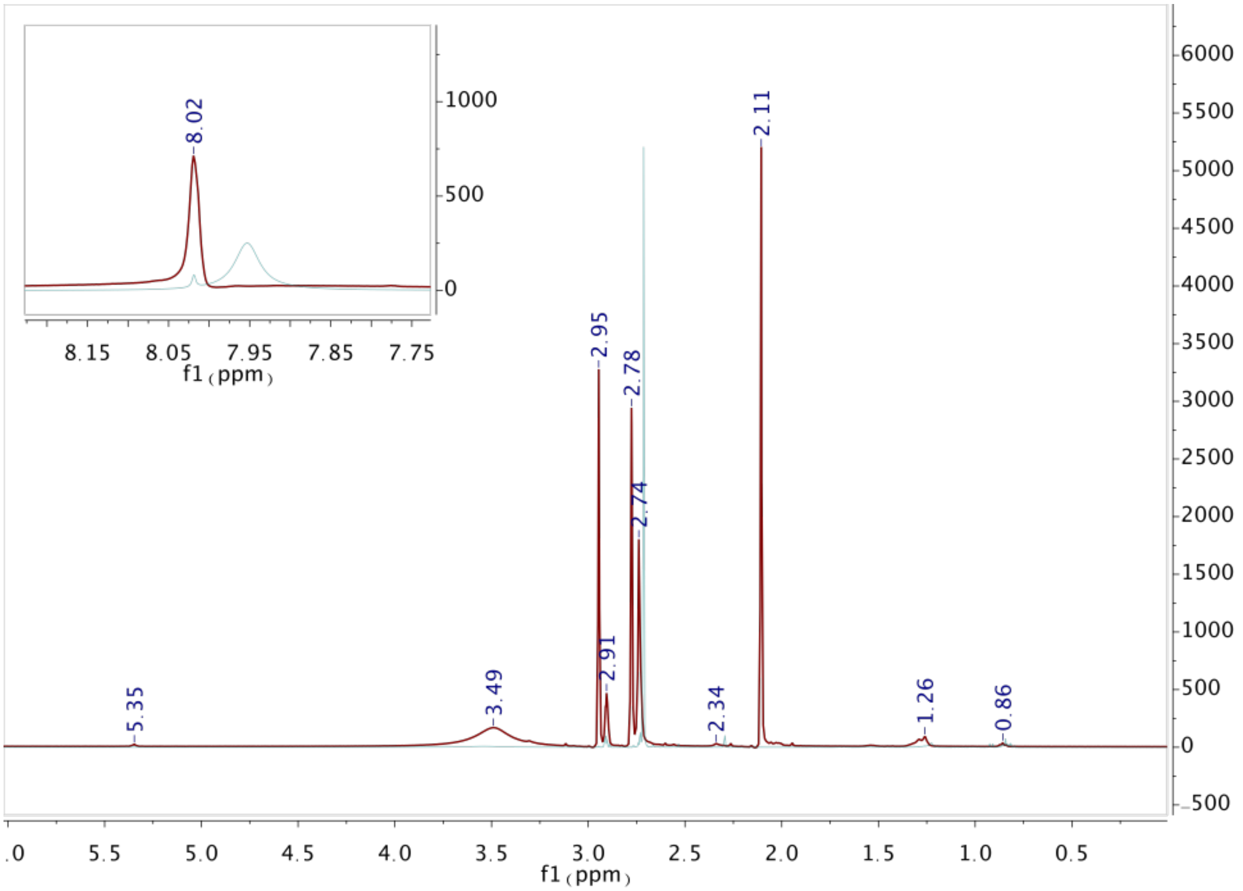

Supplementary Figure 7 | ^{1}H NMR spectra for PbI_2 + MAI (light blue peaks) and $MAPbI_3$-capped QDs (red peaks) in d_7-DMF. ^{1}H NMR spectra for PbI_2 + MAI (light blue peaks) and $MAPbI_3$-capped QDs (red peaks) in d_7-DMF. Spectra referenced to residual nondeuterated DMF peak at 8.019ppm. Peaks ∂ = 0.86, 1.26, 2.34 and 5.35 are from residual oleic acid / bound oleate. Peaks belonging to MAI are CH_3 (∂ = 2.74 ppm) and NH_3^+ (∂ = 3.49 ppm).

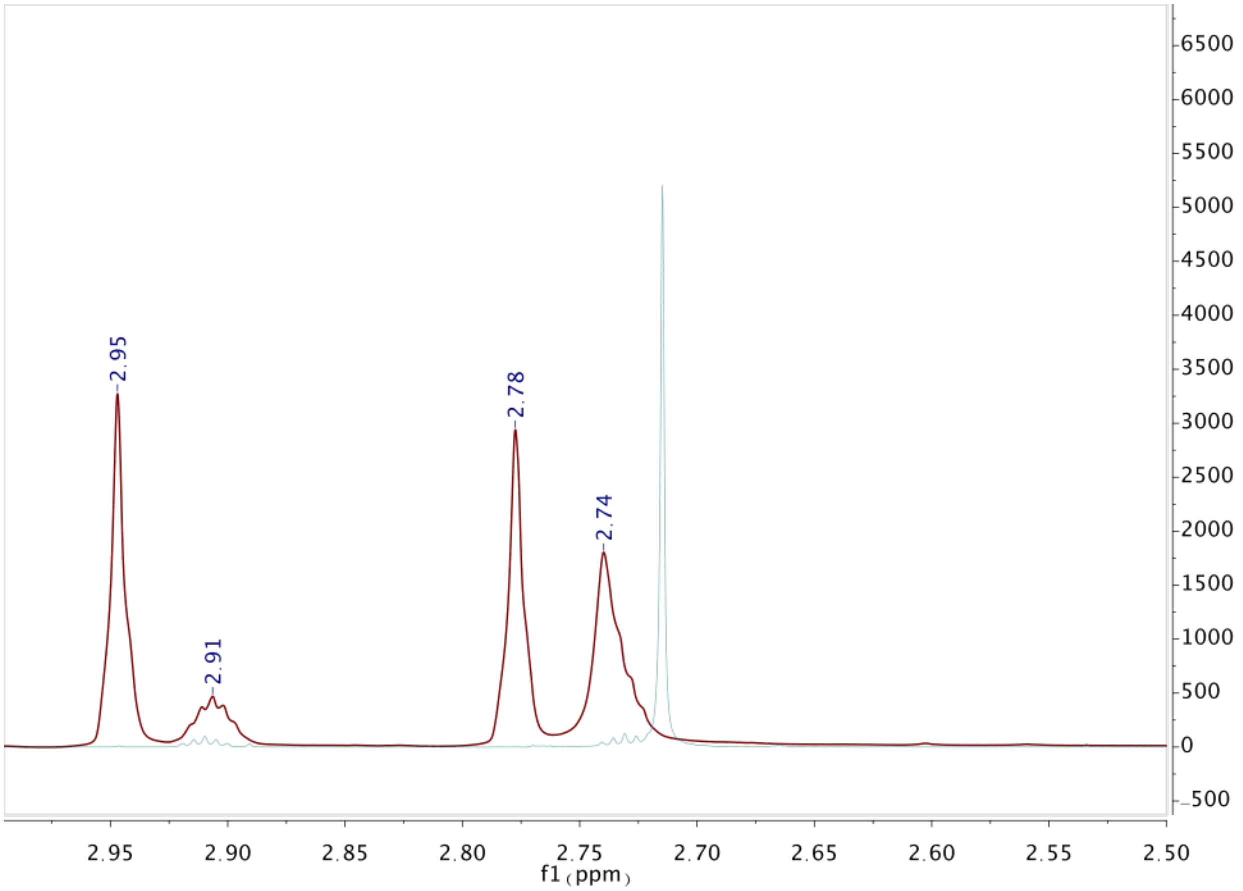

Supplementary Figure 8 | Zoom-in ^{1}H NMR (∂ = 2.50 – 3.00 ppm) of PbI_2 + MAI (blue) and $MAPbI_3$-capped dots (red). The light blue peak at ∂ = 2.71 ppm is from $–CH_3$ in free MAI, which shifts from ∂ = 2.71 ppm to 2.74 ppm when bound to QD, and broadens. Peaks at ∂ = 2.78 and 2.95 are from DMF and ∂ = 2.91 from minor side reactions that occur when PbI_2 + MAI are mixed in DMF. Solvent peaks appear much more intense for the QDs because only a small amount of QDs could be solubilized in DMF, and only the surface contains ligands giving NMR signal.

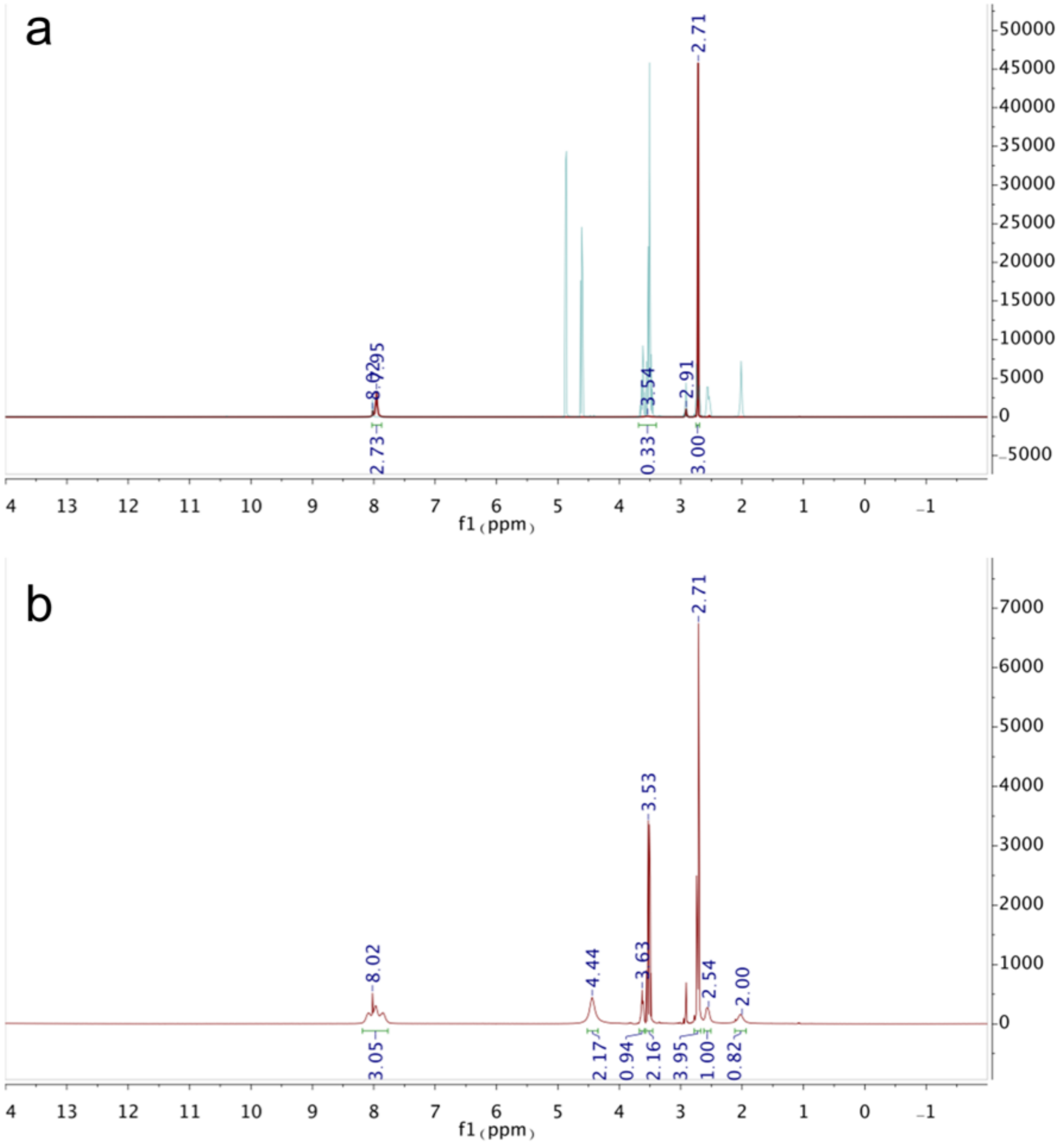

Supplementary Figure 9 | 1D ^{1}H NMR spectra of (a) stacked spectra of solution TG in DMF (light blue peaks) and solution 1:1 mixture of PbI_2 and MAI in DMF (red peaks), (b) a 1:1 mixture of 1+2. All spectra are referenced to the residual DMF peak at 8.019 ppm. Comparing the two spectra, most spectral features are conserved when TG and MAI + PbI_2 are mixed in solution. The most notable change is the two well defined OH peaks from TG (4.61 and 4.86 ppm blue peaks) becoming broadened and shifted up-field to 4.44 ppm.

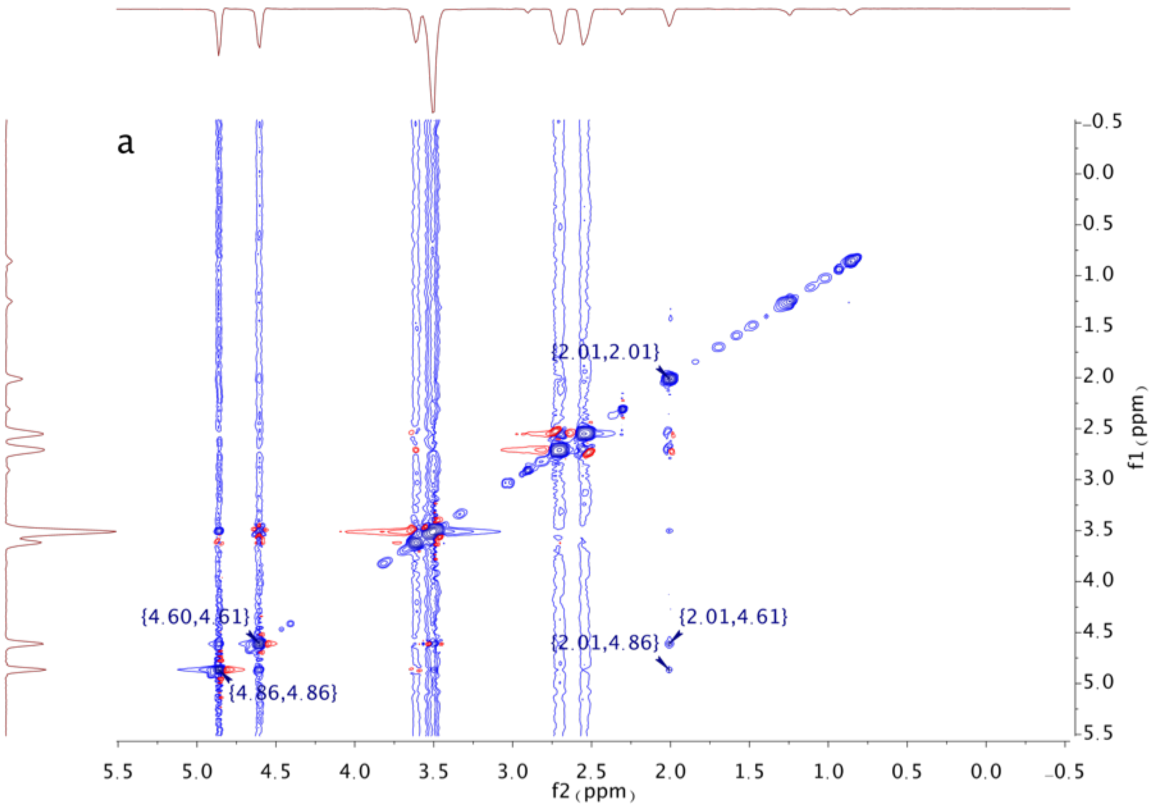

Supplementary Figure 10 | ^{1}H NOESY spectra of (a) solution 1 (b) solution 2 and (c) 1:1 mixture of 1+2. Proton peaks from ammonium cation in MAI are found at both ∂ = 8.12 and 3.72 ppm. The peaks integrate to a total of 3 in agreement with 3 protons from the $–CH_3$ protons from MAI, and cross peaks in Figure S6(b) show through-space coupling. TG hydroxyl proton peaks are located at ∂ = 4.60 and 4.86 ppm in Figure 6a, with minor cross peaks at {2.01, 4.86} and {2.01, 4.60} showing through-space coupling to the thiol proton from TG. These hydroxyl protons and the thiol proton are preserved when mixed with $MAPbI_3$ precursors in DMF, evidenced by the integration matching in Figure 5b for peaks at ∂ = 2.00 (-SH) and 4.44 ppm (x2 -OH). ^{1}H NOESY spectrum of the mixture shows the cross peak between TG hydroxyl and thiol protons is maintained at {2.01, 4.37}.

(Supplementary Figure 10 Continued)

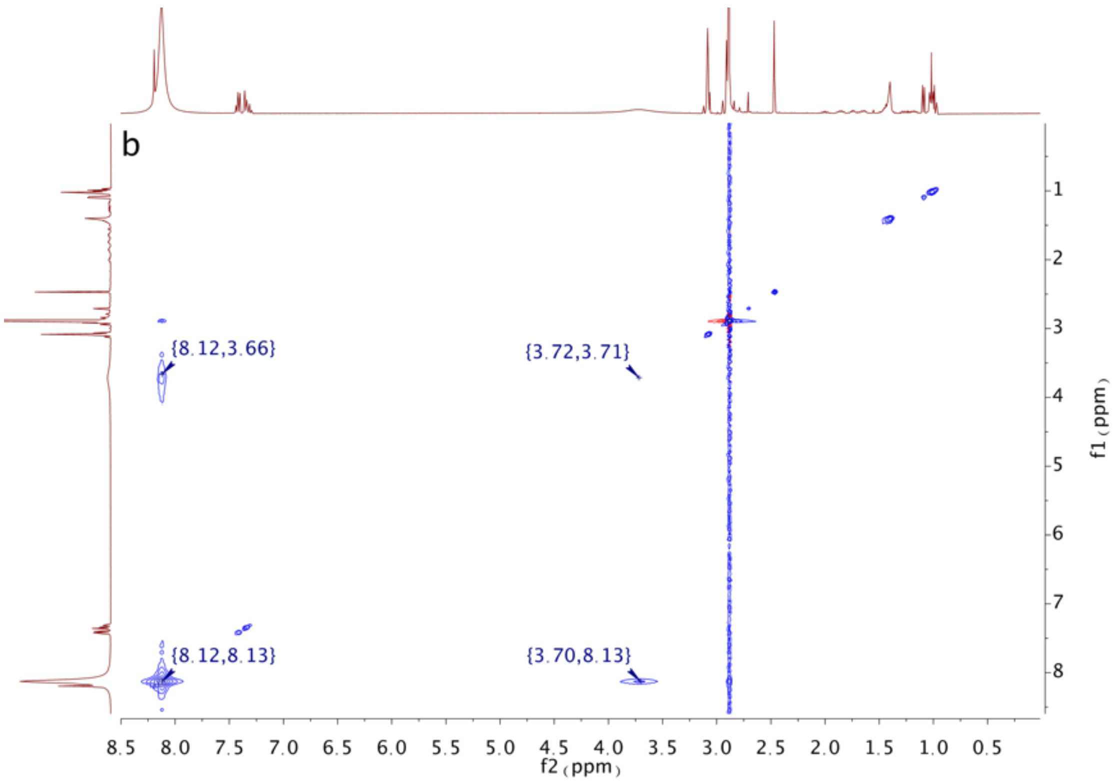

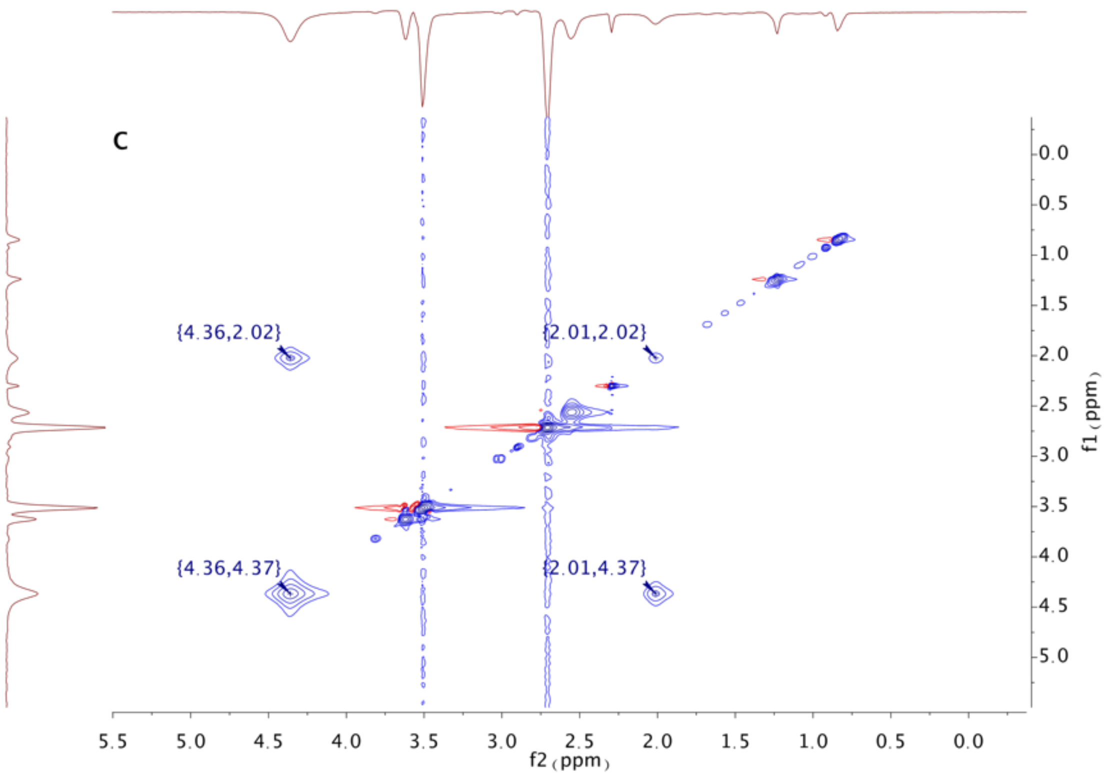

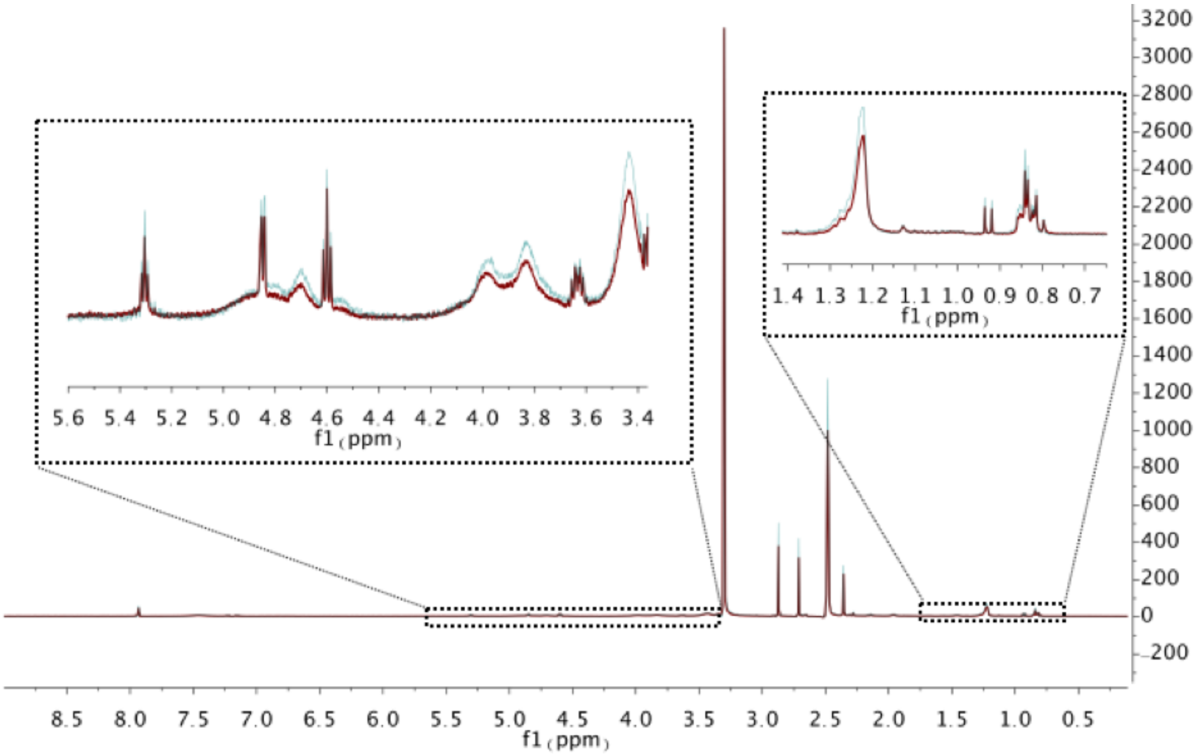

Supplementary Figure 11 | 1H NMR spectra of mixed QD sample before and after annealing process (red: sample before annealing; blue: sample after annealing).

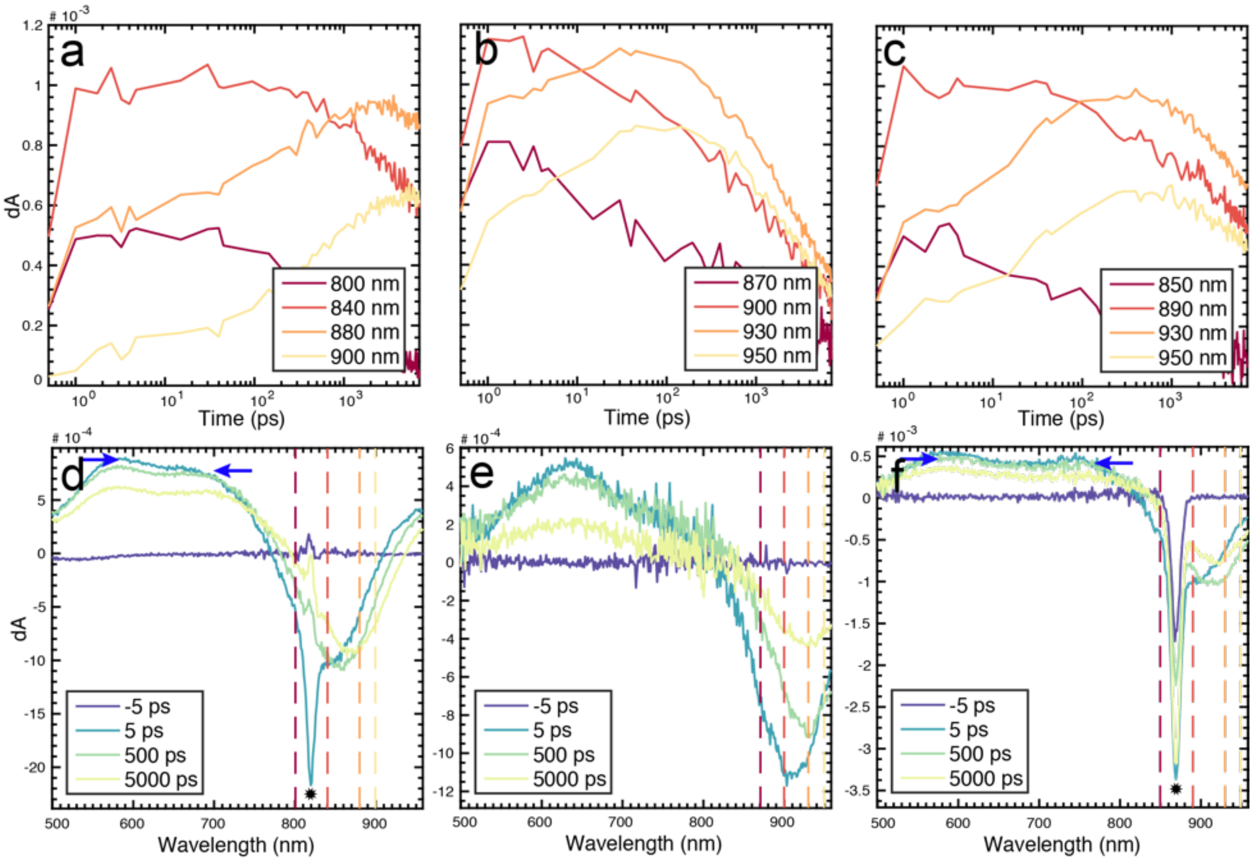

Supplementary Figure 12 | Band tailing dynamics from low power TA. (a–c) Spectral time traces and **(d–f)** wavelength traces for TG-capped QDs (a and d), $MAPbI_3$-capped QDs (b and e) and BHJ-QDs (c and f). Dashed vertical lines on Figures d, e, f indicate by color the wavelengths for the time traces in the Figures a, b, c, respectively. Blue arrows point to the two distinct ESA peaks present in the TG-capped and BHJ-QDs. The black star indicates a large peak from pump scatter, and all time traces are taken at wavelengths sufficiently far away from the scattering peak. In panel (d), the small negative signal at the scatter peak is from uneven background subtraction, and is not a real dynamical spectral feature.

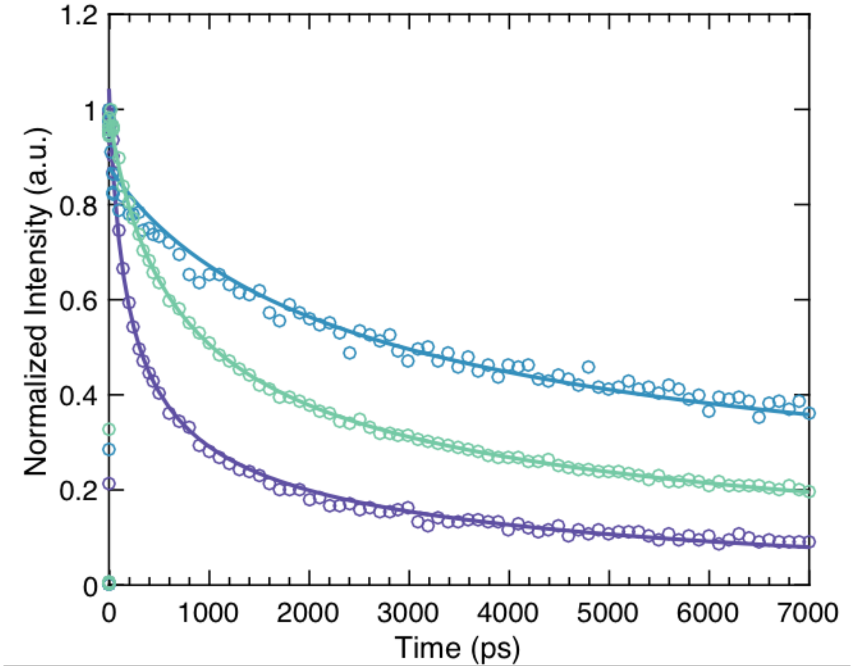

Supplementary Figure 13 | Auger dominated decay from high power TA. TG-capped QD films (blue trace), $MAPbI_3$-capped QD films (purple trace) and BHJ QD films (green trace) are all pumped with high excitation fluences to induce Auger recombination as carriers diffuse after photoexcitation.

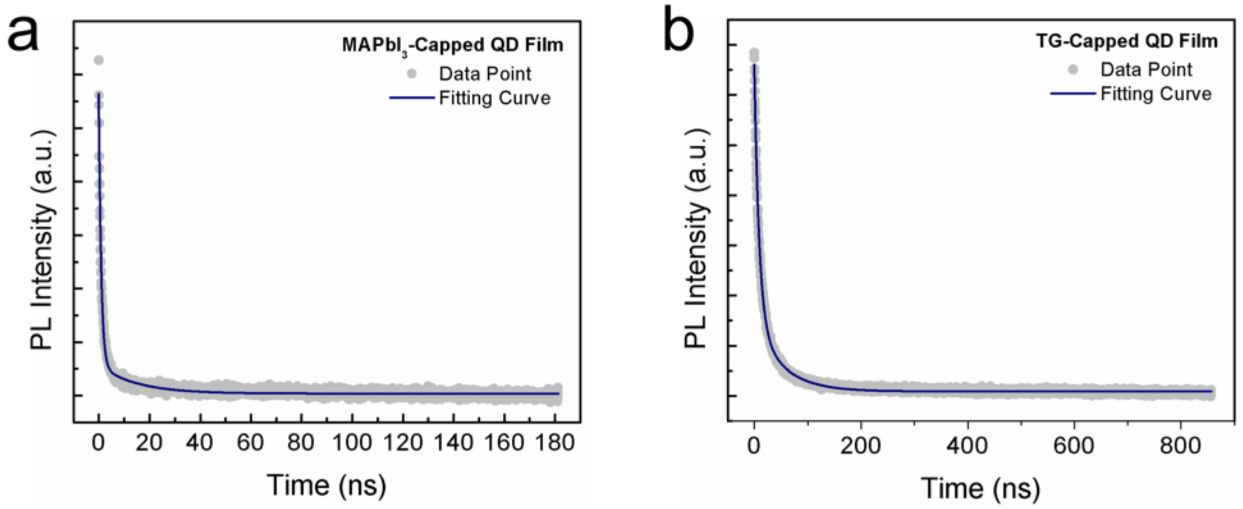

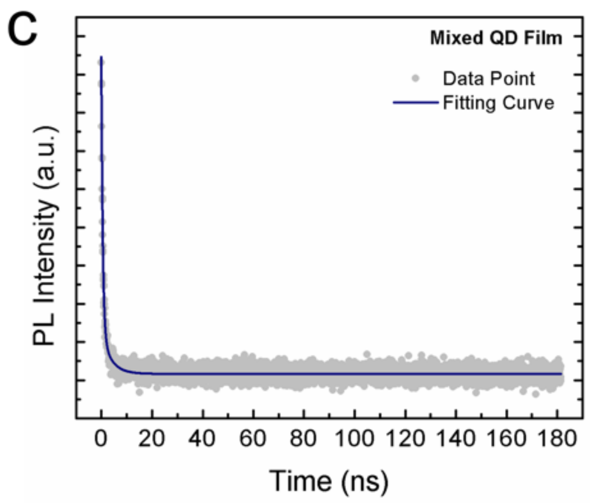

d

Type of QDs	τ_1 (ns)	τ_2 (ns)
$MAPbI_3$-QDs	0.95	14.76
TG-QDs	8.91	51.10
Mixed QDs	0.58	3.67

Supplementary Figure 14 | Time-resolved PL decays of QD films. **(a)** $MAPbI_3$-passivated QDs. **(b)** TG-passivated QDs. **(c)** Mixed QD film. **(d)** Summary of time-resolved PL nanosecond components lifetime for three types of QDs.

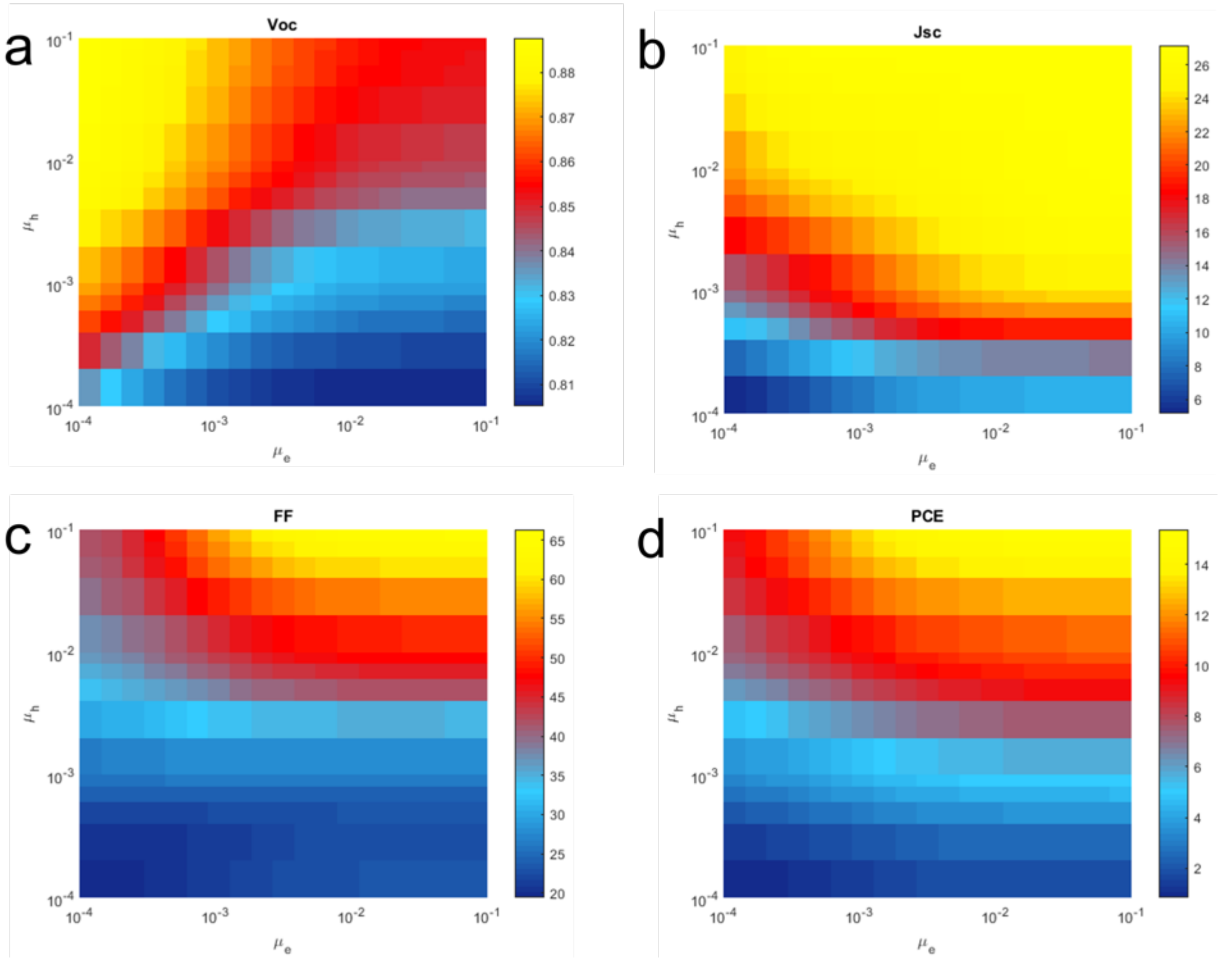

Supplementary Figure 15 | Optoelectronic simulations modeling the impact of balanced extraction in the photovoltaic performance: **(a)** Open-circuit voltage; **(b)** short-circuit current; **(c)** fill-factor; and **(d)** power conversion efficiency. Details on the simulations can be found in Table S3.

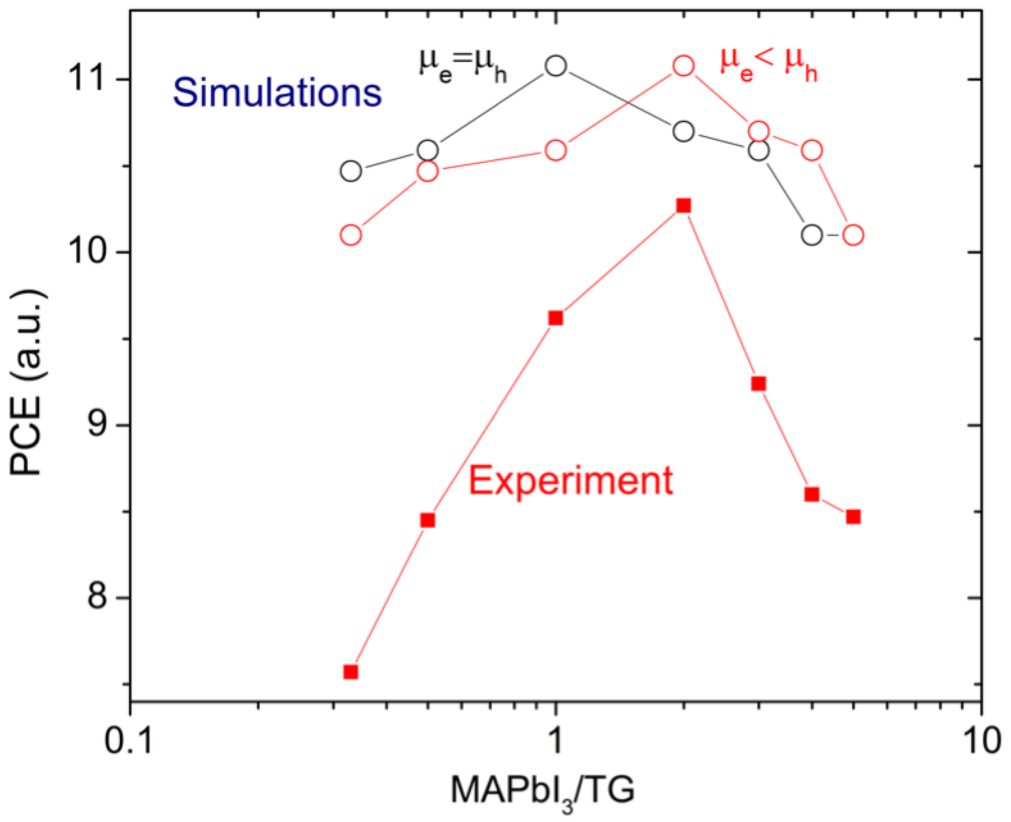

Supplementary Figure 16 | Comparison of experimental and simulation results.

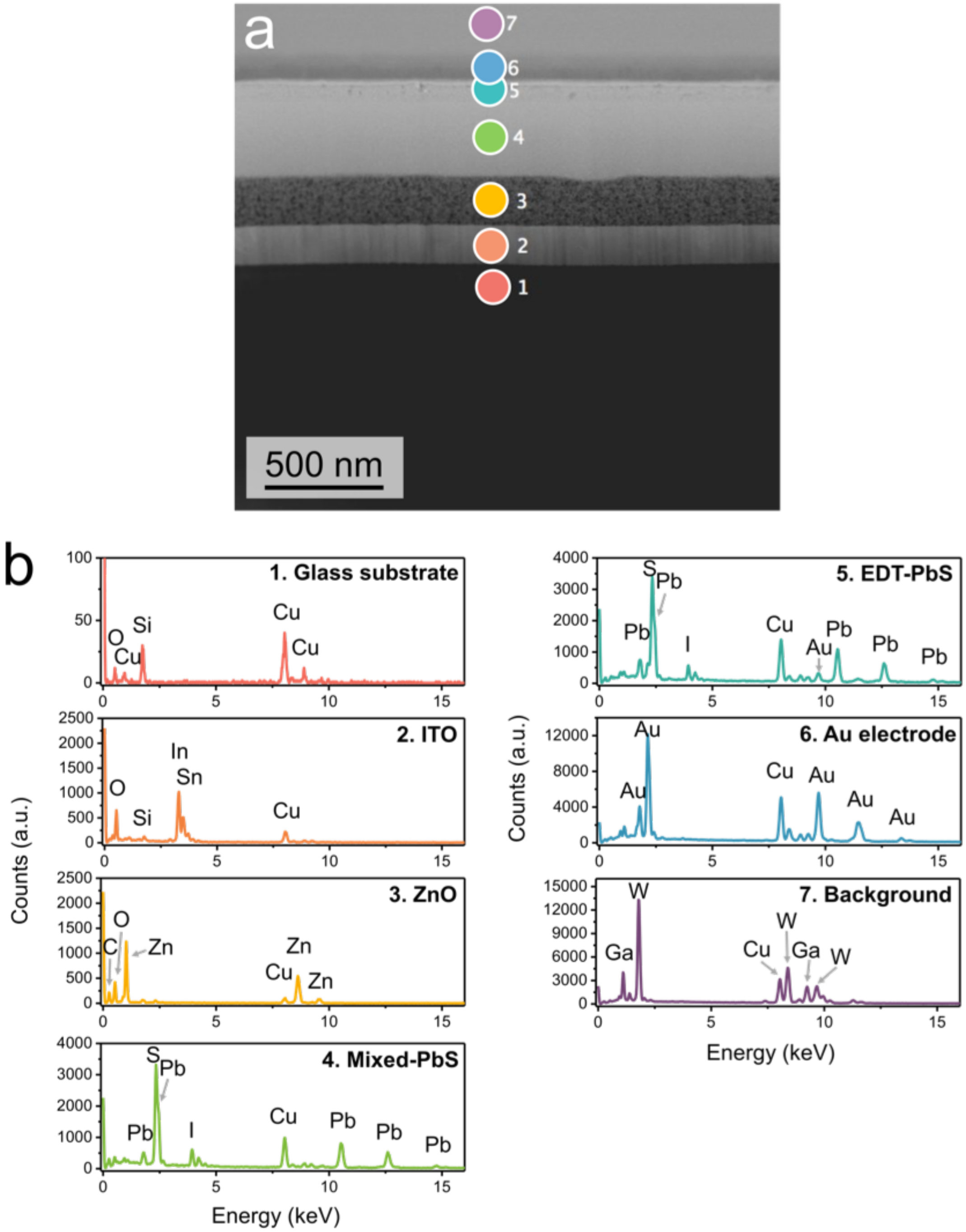

Supplementary Figure 17 | EDX analysis of FIB processed MQD based photovoltaic device. **(a)** FIB TEM image of a mixed-quantum-dot device; **(b)** EDX results of each component highlighted in (a). Background signals include electron-beam deposited tungsten, gallium from FIB process, and copper from the copper TEM grid.

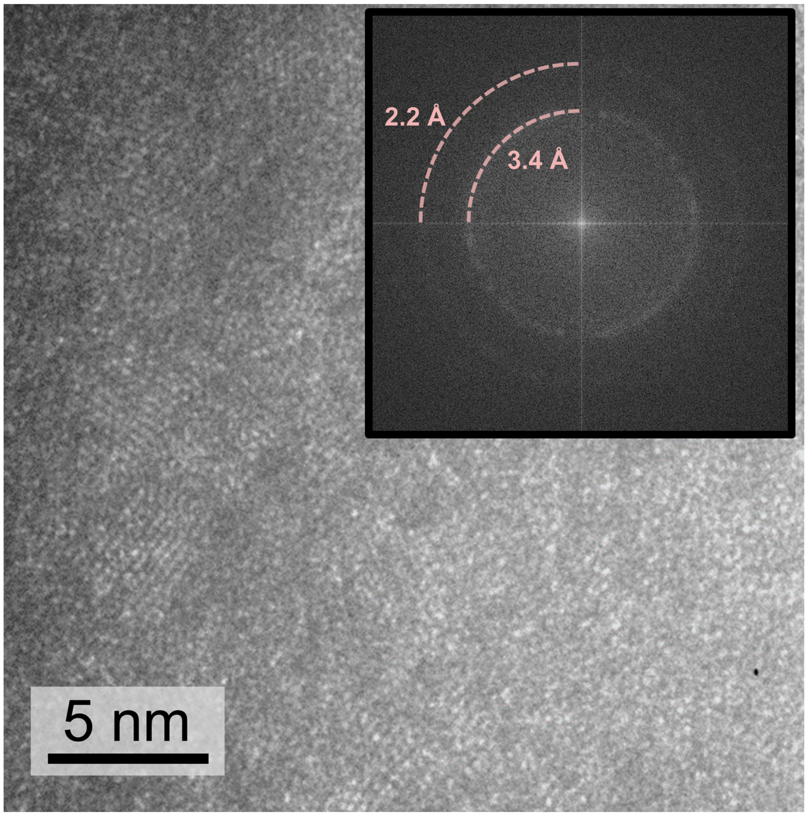

Supplementary Figure 18 | Electron microscopy analysis of FIB processed MQD solid solution film. Bright-field TEM image of focused ion beam (FIB) processed mixed QD film (thickness around 100 nm) showing random PbS lattice orientation on closely packed QDs. Inset: FFT pattern showing d-spacing patterns assigned to PbS {111} (3.4 Å) and {220} (2.2 Å) lattice planes.

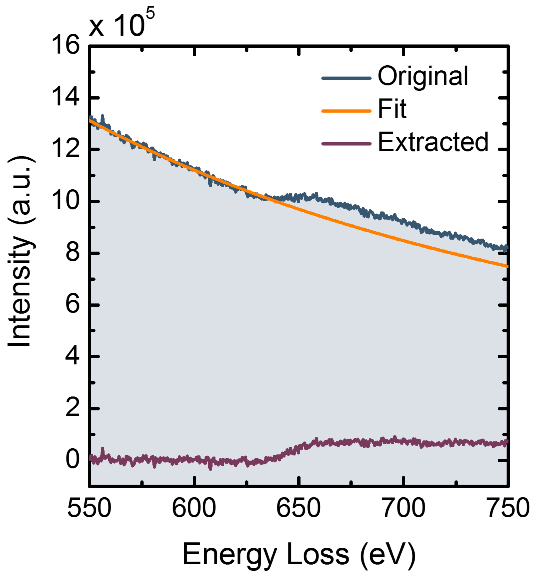

Supplementary Figure 19 | EELS spectrum of iodine component across the QD active layer shown in Fig. 4d. The major edge of iodine $M_{4,5}$ signal starts at around 625 eV.

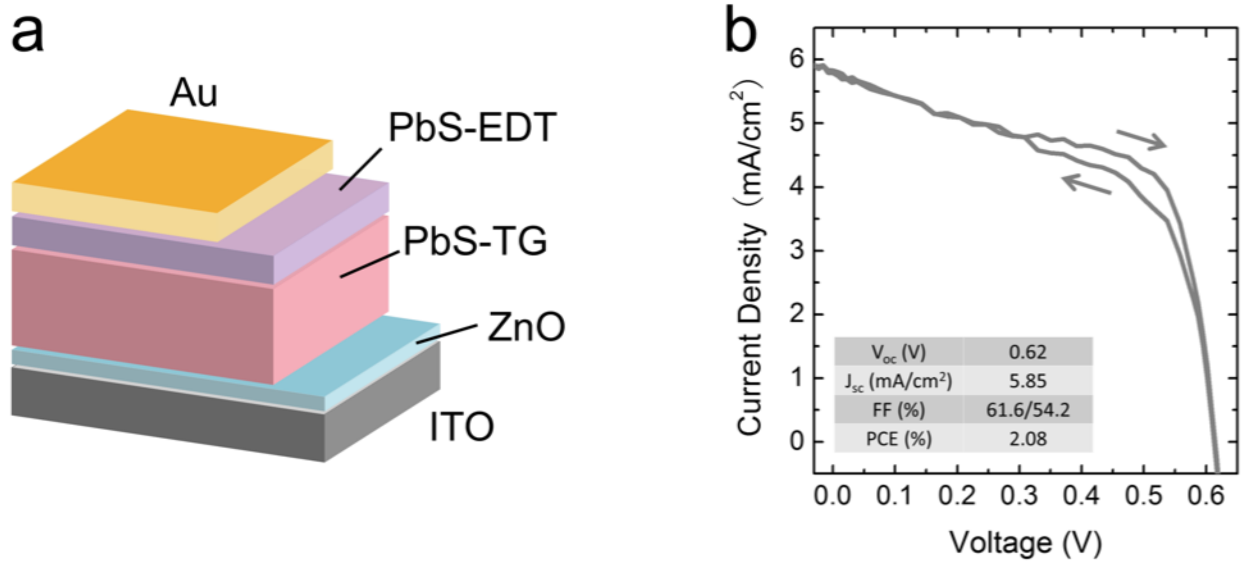

Supplementary Figure 20 | TG-passivated PbS QD photovoltaic device architecture and the best performance. (a) Schematic of the device structure. **(b)** *J*-*V* characteristics under simulated AM 1.5 illumination

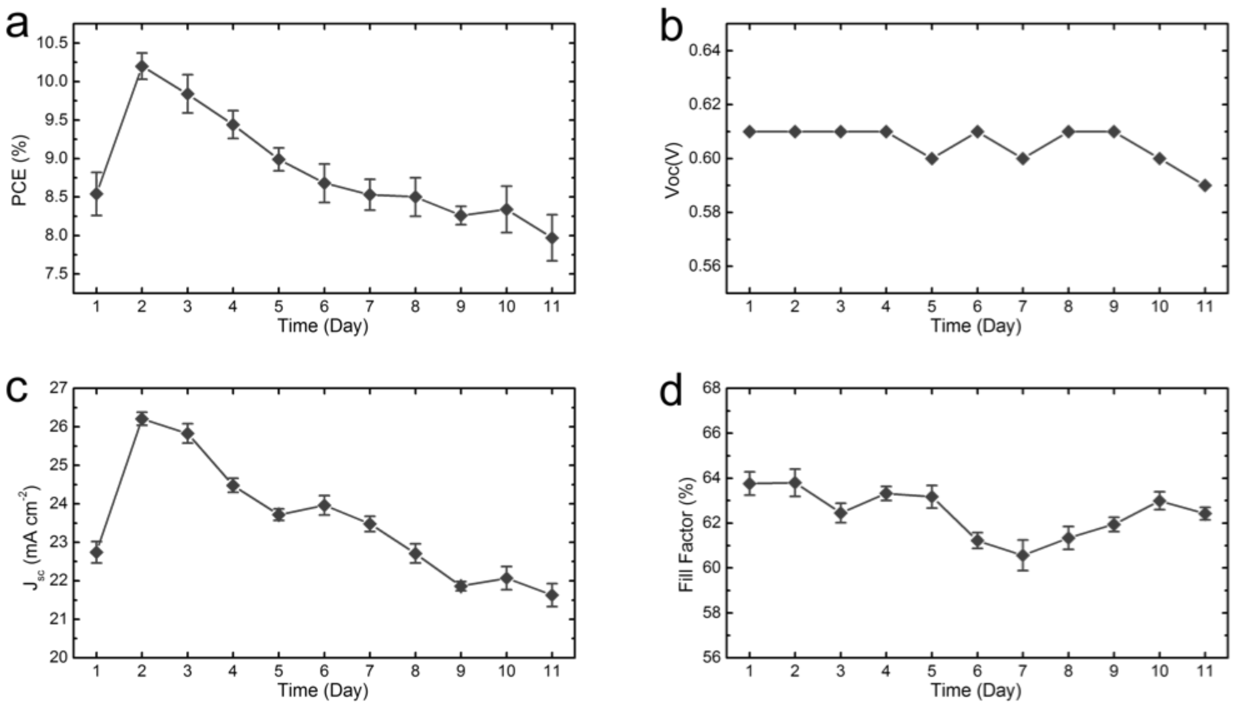

Supplementary Figure 21 | Stability test for the best devices stored in air (A:D ratio = 2:1): **(a)** Overall PCE, **(b)** V_{oc}, **(c)** J_{sc}, and **(d)** fill factor. Error bars represent the standard deviation of several devices.

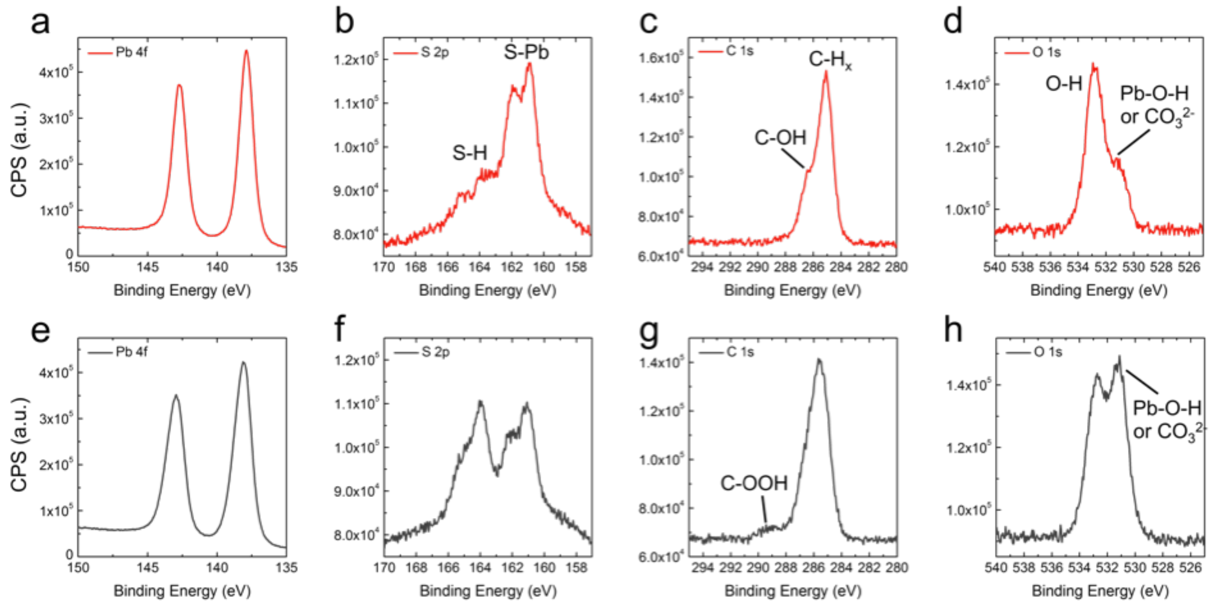

Supplementary Figure 22 | High resolution XPS results of different elemental regions of annealed TG-capped PbS QD film stored in nitrogen (a-d) and air (e-h) for 3 days, respectively: (a, e) lead 4d; **(b, e)** sulfur 2p; **(c, g)** carbon 1s; **(d, h)** oxygen 1s. Surface oxidation of TG-capped PbS QDs is reflected on the decrease of the S-Pb signal at ~161 eV (S 2p) and the increase of oxidation signal at 531.3 eV (O 1s).[29, 30]

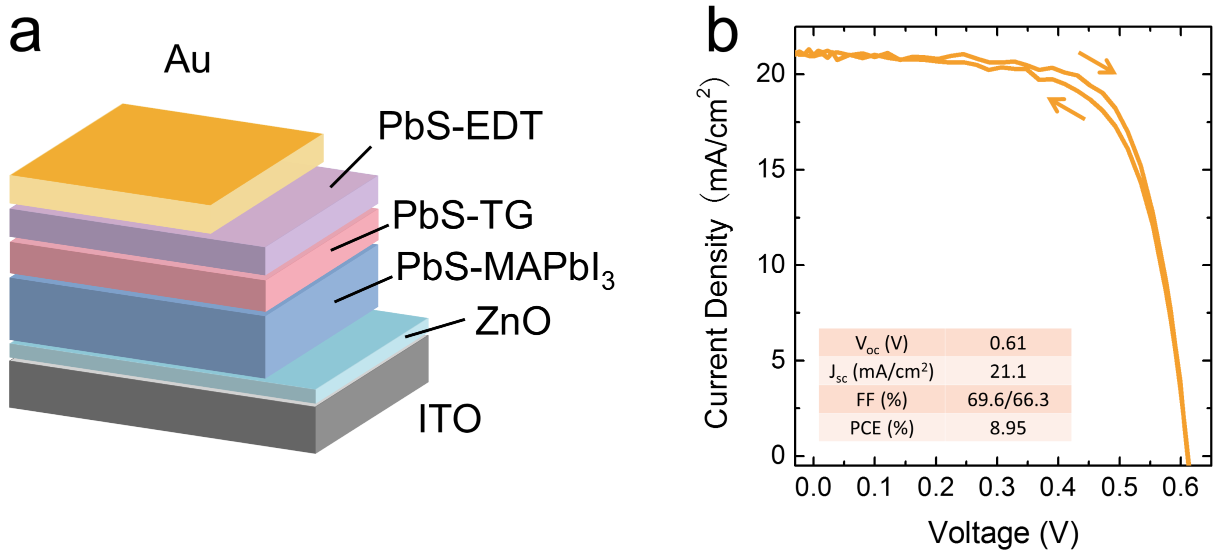

Supplementary Figure 23 | TG-PbS/MAPbI$_3$-PbS bilayer photovoltaic device architecture and the best performance. (a) Schematic of the device structure. **(b)** *J-V* characteristics under simulated AM 1.5 illumination.

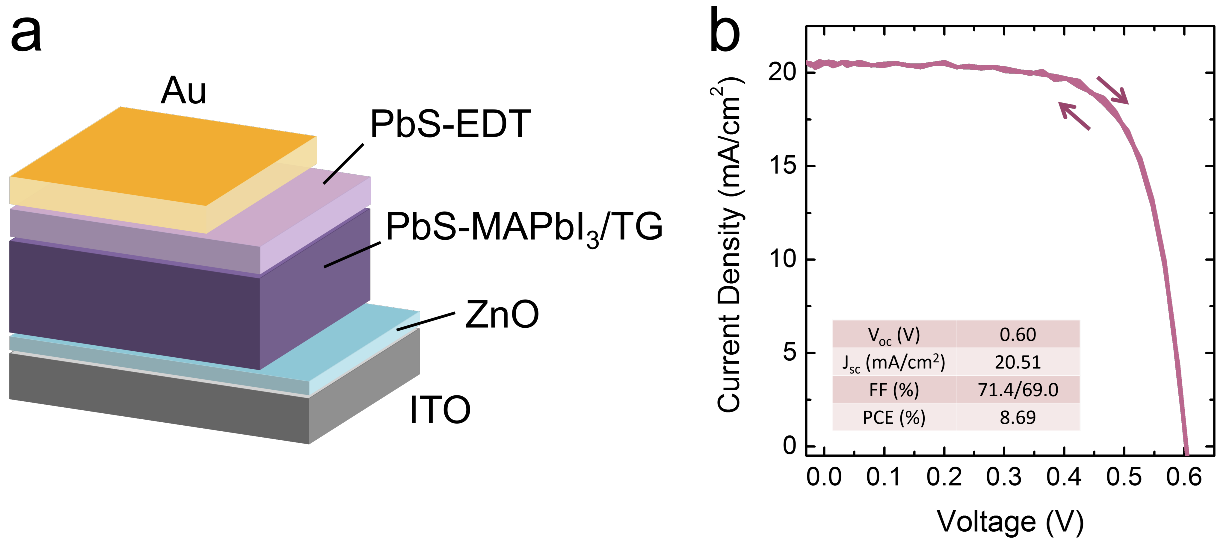

Supplementary Figure 24 | MAPbI$_3$/TG co-treated QD photovoltaic device architecture and the best performance. (a) Schematic of the device structure. **(b)** *J-V* characteristics under simulated AM 1.5 illumination.

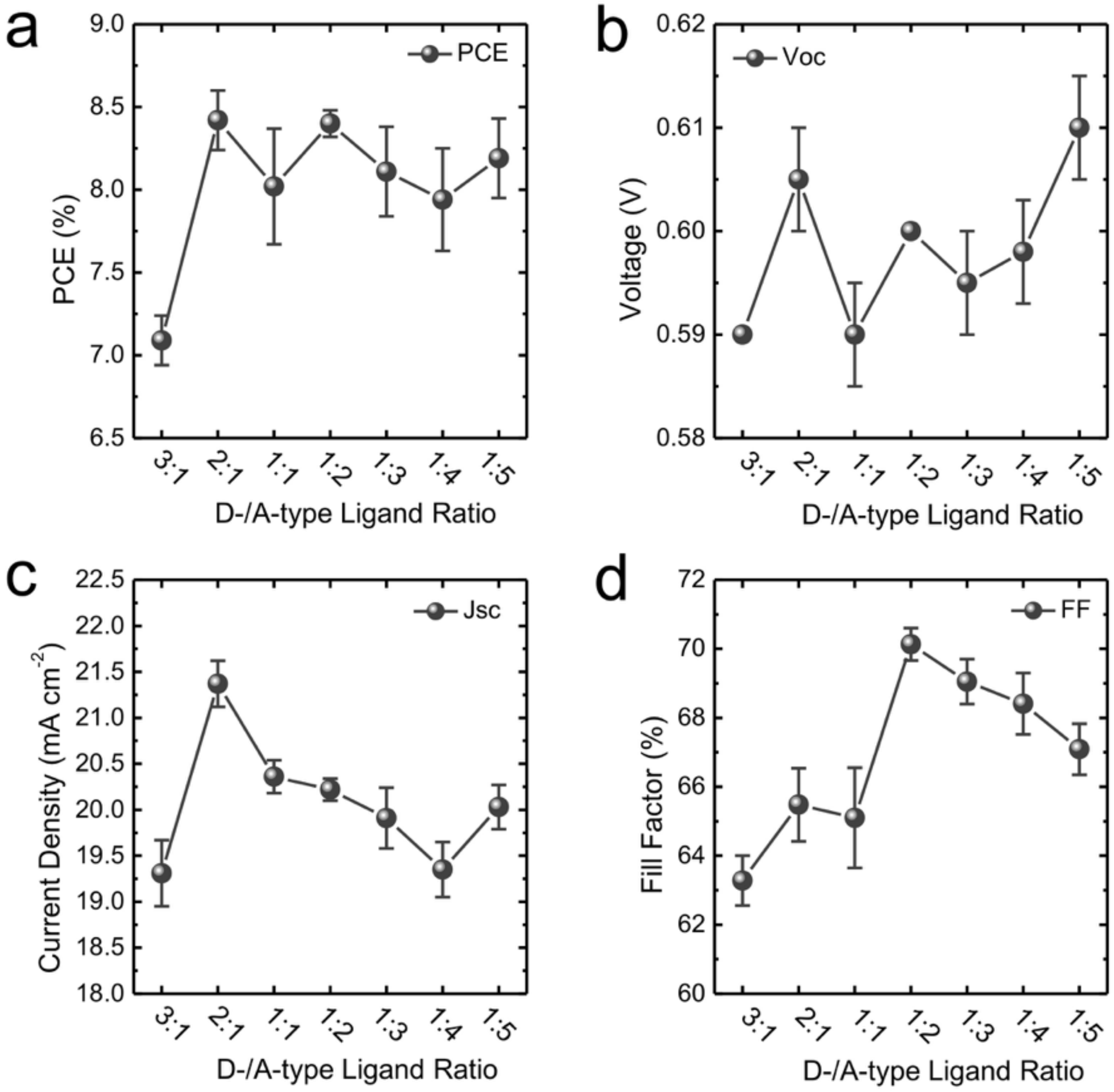

Supplementary Figure 25 | Solar cell performance of $MAPbI_3$/TG co-treated QD photovoltaic device with various D- (TG)/A- ($MAPbI_3$) type ligand ratios: (a) Overall PCE, **(b)** V_{oc}, **(c)** J_{sc}, and **(d)** fill factor. Error bars represent the standard deviation of several devices.

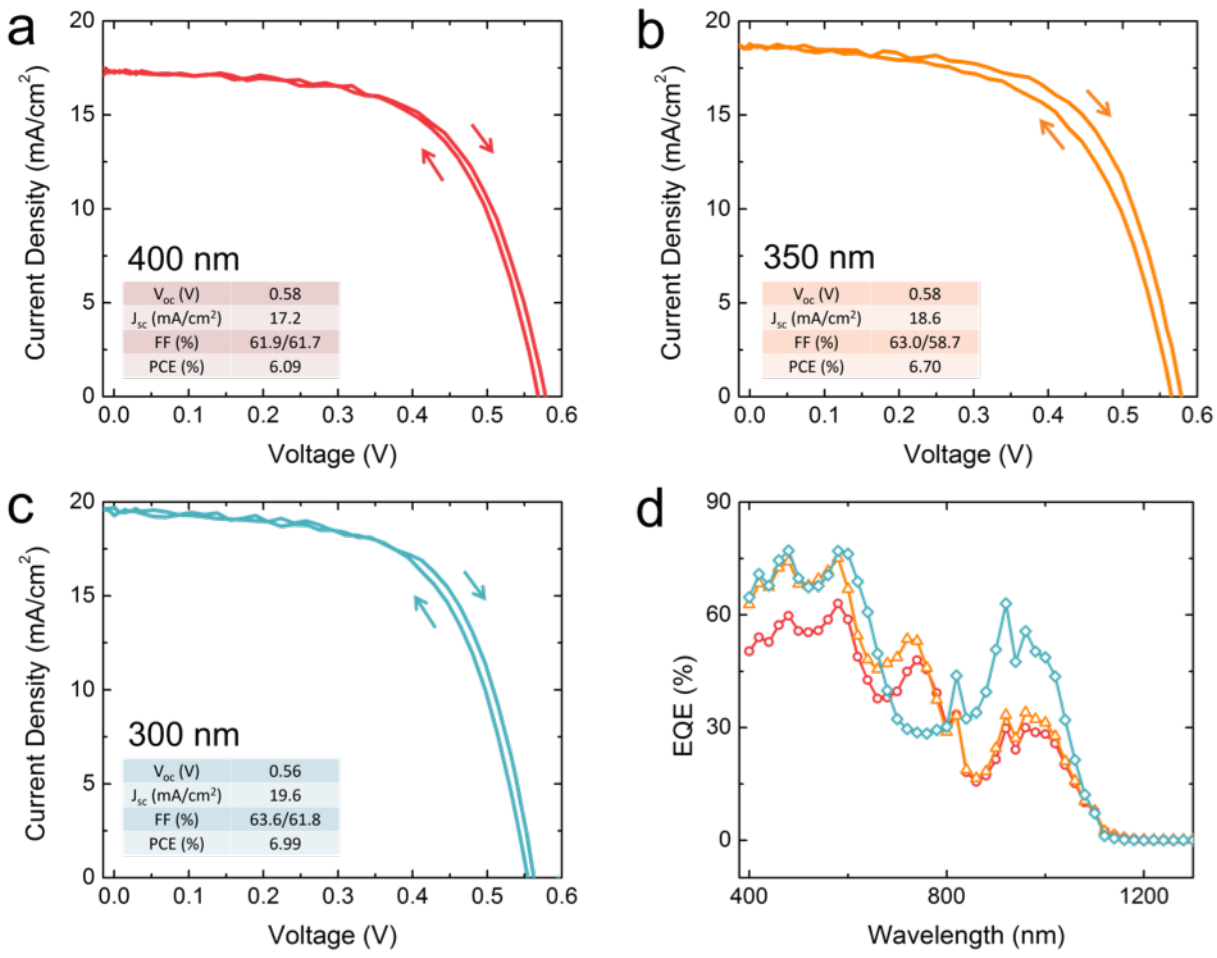

Supplementary Figure 26 | Current−voltage (*J*−*V*) characteristics under simulated AM 1.5 illumination for control devices (architecture shown in Figure S22a) with different thickness of $MAPbI_3$/TG co-treated PbS layer: **(a)** 400 nm, **(b)** 350 nm, and **(c)** 300 nm. **(d)** EQE spectra of these three devices.

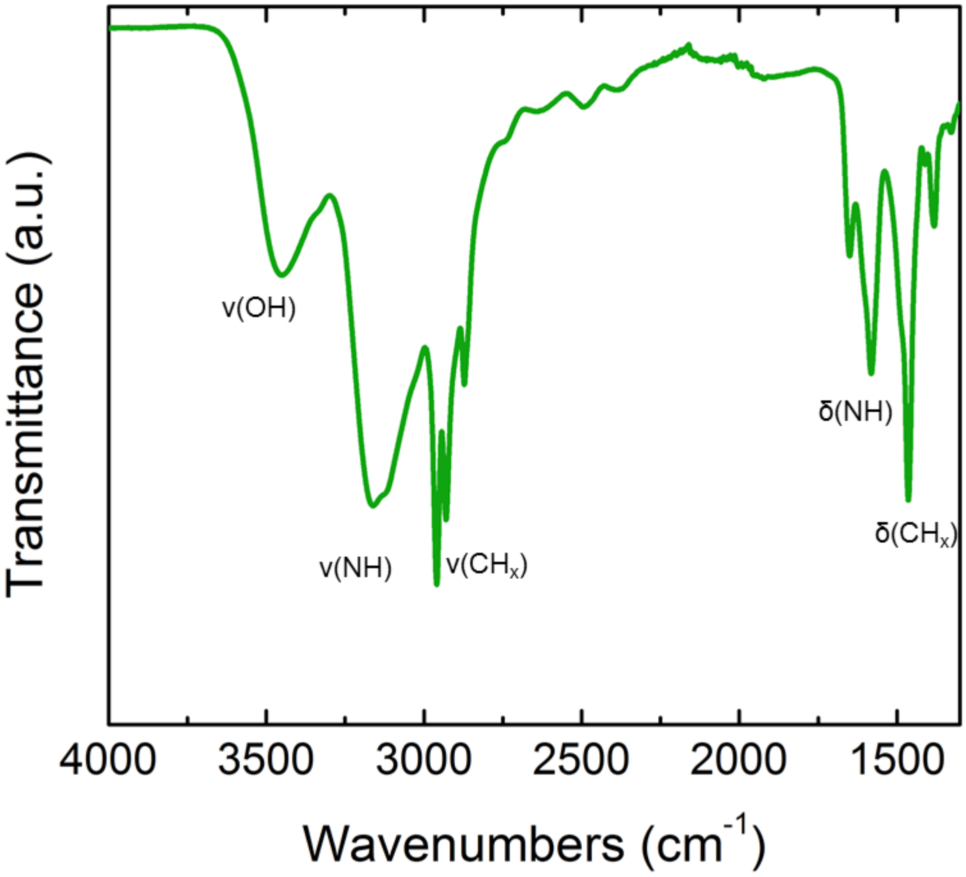

Supplementary Figure 27 | FT-IR spectra of co-treated QD: Characteristic FT-IR peak signals and intensities such as the ν(-OH) (3400–3600 cm^{-1}), ν(N-H) (3000–3300 cm^{-1}), ν(-CH_x) (2850–2950 cm^{-1}), δ(NH) (1550–1700 cm^{-1}), and δ(CH_x) (1380–1460 cm^{-1}) match closely to those of mixed QD films.

Supplementary Table 1 | Simulated energy levels of highest valence band (VB) and lowest conduction band (CB) of various types of surface-passivated PbS QDs.

QD Type	Highest VB (eV)	Lowest CB (eV)
TG-capped PbS	-5.2	-3.9
$MAPbI_3$-capped PbS	-5.6	-4.3
EDT-capped PbS	-5.2	-3.9

Supplementary Table 2 | UPS results of the energy levels of highest VB, lowest (CB) and work function of TG- and $MAPbI_3$-capped PbS QDs.

QD Type	Highest VB (eV)	Lowest CB (eV)	Work Function (eV)
TG-capped PbS	-5.13	-3.82	-4.22
$MAPbI_3$-capped PbS	-5.64	-4.33	-4.96

Supplementary Table 3 | Fitting parameters for high power TA measurements

**Ligand**	**TG**	**$MAPbI_3$**	**BHJ**
k	1E-06	1.054E-04	3.53E-05
c	0.0004588	0.004778	0.001352

Fitting parameters from Table S2 show the intermediacy of the BHJ film dynamics compared to the TG- and $MAPbI_3$-capped QD films. Different rates of Auger recombination reflect the mobilities of the different films found in the low power TA experiments (i.e., slower Auger occurs for films with lower mobilities since carriers do not find each as fast to recombine).

Supplementary Table 4 | Details of SCAPS simulation used parameters. The Mixed QD was simplified by a homogeneous effective intrinsic material.

	**EDT layer**	**MQD layer**	**ZnO layer**
Thickness (nm)	70	350	125
Bandgap edge (eV)	1.2	1.2	3.2
Electron affinity (eV)	3.9	4.160	4.3
Permittivity (er)	20	20	66
CB/VB DOS (cm^{-3})	1E19	1E19	1E19
Electron mobility (cm^2/Vs)	2E-4	swept	5E-2
Hole mobility (cm^2/Vs)	2E-4	swept	5E-2
Electron/hole thermal velocity (cm/s)	7E3	7E3	1E7
Ndonor (cm^{-3})	1E14	5E16	1E17
Nacceptor (cm^{-3})	1E17	5E16	0
EDT/TBAI defect (neutral)			
Capture cross section (cm^2)	1.2E-13	1.2E-13	
Position below Ec (eV)	0.3	0.3	
Density (cm^{-3})	5E16	1E16	
TBAI-ZnO interface defects (neutral)			
Capture cross section (cm^2)	1E-19		
Position above Ev (eV)	0.6		
Density (cm^{-3})	1E16		

Supplementary Note 1 | Explanation of TA Spectroscopy: TA spectroscopy is sensitive to excited states of photogenerated excitons and carriers, where an ultrashort pulse excites the sample, and then a white light continuum probe of variable time delay probes the dynamics. TA is a pump-probe technique, where spectra are generated by taking the difference of the probe transmission spectrum with the 'pump on' and the 'pump off'. After photoexcitation of some percentage of the ground state population into the excited state, this transition is no longer accessible for the probe pulse, meaning more probe light passes through for 'pump on' compared to 'pump off'. The differential transmission spectrum for $T_{pump\text{-}off}$ - $T_{pump\text{-}on}$ (usually written as $\Delta T/T$) then shows a negative *bleaching* signal at the wavelength for that transition. Similarly, when photoexcited carriers can be further excited to a higher excited state by the probe pulse, this transition is only possible following the pump pulse, meaning the 'pump on' while allow less probe light through at this transition than 'pump off'. Therefore, there are positive *excited state absorption (ESA)* signals in the $\Delta T/T$ spectrum. $\Delta T/T$ spectra are generated at each time delay between pump and probe pulses, and so the decay of bleaching and ESA signals tracks the relaxation of excited states back to the ground state, or energy / charge transfer processes as new signals may arise after time zero.

Bandtailing is the effect of carriers finding the lowest energy sites in a QD solid by diffusion from higher to lower energy states. In Figure S10, the bandtailing is seen to be most rapid the $MAPbI_3$-capped QDs, slowest in the TG-capped QDs, and intermediate for the BHJ-QDs. Spectroscopically, bandtailing is observable as spectral diffusion, where the bleaching peak from the initial photogenerated population redshifts and narrows with time as the carriers find low energy sites in the inhomogeneous distribution. The rate of spectral diffusion is then linked to carrier mobility. Siebbeles et al.[21] have reported similarly rapid spectral diffusion and

mobilities before in PbSe solids, also measured by taking time traces on the higher and lower energy sides of the QD bleach peak in TA spectra.

The intermediate spectral diffusion rate in the BHJ film indicates that the dynamics in the mixed film are a superposition of those of TG and $MAPbI_3$. This is supported by the qualitatively similar ESA signals seen in the TG-capped and BHJ films but not the in $MAPbI_3$-capped films. The dynamics of the BHJ film cannot be mostly due to TG-capped QDs since the mobility is far high than the TG film, thus the retention of the ESA features indicates that a portion of the film is TG-capped QDs, while the much more rapid mobility is due to the $MAPbI_3$-capped QDs.

Supplementary Note 2 | Data fitting of Supplementary Figure 13: Data from Auger experiments are fitted to the equation:

$$\frac{dn(t)}{dt} = -kn(t) - cn^3(t) \qquad \text{(Supplementary Equation 1)}$$

Where the linear term with coefficient *k* accounts for normal radiative recombination, and the 3rd order term with coefficient *c* accounts for Auger recombination, which is a three-body mechanism.

Supplementary References:

29 Laajalehto, K., Smart, R. S. C., Ralston, J., & Suoninen, E. STM and XPS investigation of reaction of galena in air. *Appl. Surf. Sci.* **64**, 29-39 (1993).

30 Jo, C. H. et al. Low-temperature annealed PbS quantum dot films for scalable and flexible ambipolar thin-film-transistors and circuits. *J. Mater. Chem. C* **2**, 10305-10311 (2014).
